# Catalyst Functionalization for Elevating Performance in Ammonia Protonic Ceramic Fuel Cells via Relay Thermo‐Electrocatalysis

**DOI:** 10.1002/EXP.20240431

**Published:** 2026-02-10

**Authors:** Huihuang Fang, Zefeng Wang, Jiangping Chen, Jiacheng You, Yiting Jiang, Puxin Yang, Qian Lin, Haoyu Zhang, Fulan Zhong, Yu Luo, Lilong Jiang

**Affiliations:** ^1^ National Engineering Research Center of Chemical Fertilizer Catalyst (NERC‐CFC) School of Chemical Engineering Fuzhou University Fuzhou Fujian China; ^2^ Qingyuan Innovation Laboratory Quanzhou Fujian China

**Keywords:** ammonia decomposition, ammonia, catalyst, proton ceramic fuel cells, thermo‐electrocatalysis

## Abstract

Direct ammonia proton ceramic fuel cell is one of the most attractive strategies for ammonia to power at intermediate temperatures (400°C–600°C). Yet, it still remains greatly challenging, involving complex and sluggish processes, such as ammonia oxidation, ammonia decomposition (ADR), and hydrogen oxidation reaction (HOR), which always proceeds with high overpotential and low current density. Herein, we adopt the relay thermo‐electrocatalysis strategy via catalyst functionalization for enhancing the performance of NH_3_‐PCFCs, where ammonia undergoes ADR at the catalytic layer and then enters the anode to undergo HOR. The strategy not only promotes the performance of NH_3_‐PCFCs (in the case of H_2_ and NH_3_) but also enhances the absolute performance in the same fuel gas compared with bare cells. The Ru/CZ4 was synthesized for catalyst functionalization for enhanced NH_3_‐PCFCs and a general principle for designing well‐matched catalysts was proposed. Indeed, the peak power density (PPD) of Ru/CZ4 cell achieves 615 and 576 mW cm^−^^2^ for using H_2_ and NH_3_, respectively, which are 1.8‐ and 2.0‐fold higher than bare cells (327 and 283 mW cm^−^^2^ for using H_2_ and NH_3_). Additionally, the ammonia‐to‐hydrogen PPD ratio reaches to 93.7%, revealing the superior performance in both H_2_ and NH_3_ fuels. Furthermore, the detailed experiments and discussion were conducted to gain insight into the incorporated anode for the superior performance of NH_3_‐PCFC. This research offers valuable insights into the structural design and performance optimization of solid oxide fuel cells using hydrogen‐rich fuels.

## Graphical abstract

Direct ammonia proton ceramic fuel cell (NH_3_‐PCFC) typically underperforms at intermediate temperatures due to slow ammonia decomposition at the anode. By applying a Ru/CZ4 catalytic layer, this study enhances ammonia decomposition, with NH_3_‐PCFC power density at 650°C reaching 93.7% of H_2_‐fueled PCFC, offering valuable insights into optimizing fuel cell performance with hydrogen‐rich fuels.

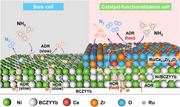

## 1 Introduction

Hydrogen (H_2_) has been considered as an ideal energy vector, yet its storage and transportation issues present significant challenges for large‐scale applications [1, 2, 3, 4, 5, 6]. In contrast to hydrogen liquefaction and solid hydrogen storage, hydrogen‐rich chemicals serve as efficient hydrogen carriers and gain great attention due to their flexible “green power–chemical power” energy cycle [7]. Among these carriers, ammonia stands out as particularly attractive due to its carbon‐free nature and established synthesis/storage infrastructures [8, 9, 10, 11, 12]. Utilizing ammonia as an energy source strongly supports the envisioned “hydrogen economy” through an evolving “ammonia economy” [12, 13].

Direct ammonia proton ceramic fuel cell (NH_3_‐PCFC) is one of the most promising strategies for ammonia utilization, which can efficiently convert chemical energy of NH_3_ into electricity at intermediate temperatures (400°C–600°C) [14, 15]. The direct use of ammonia as fuel in a typical PCFC couples the endothermic nature of ammonia decomposition reaction (ADR) and exothermic nature of hydrogen oxidation reaction (HOR) at anodes, achieving superior theoretical power efficiency [16]. Interestingly, the anodes in PCFC consist of Ni and ceramic oxides (e.g. Ni‐BaZr_0.1_Ce_0.7_Y_0.1_Yb_0.1_O_3_, Ni‐BCZYYb), which are regarded as active composites in ammonia decomposition [17, 18, 19]. It is also worth noting that the ammonia decomposition demonstrates a well match in reaction temperatures with typical anode‐supported PCFCs, providing potential in catalyst design for enhancing the performance of NH_3_‐PCFCs [20].

Compared with typical H_2_‐PCFC, the NH_3_‐PCFCs involve complex processes such as ammonia oxidation (AOR), ADR and HOR. The AOR is a kinetically sluggish process, which always proceeds with high overpotential, and low current density. Alternatively, the ammonia decomposes into dihydrogen and dinitrogen, followed by HOR at the same anode, which has great potential to achieve high performance in NH_3_‐PCFCs [21]. Nevertheless, the particle size of Ni composites at PCFC anodes is extremely large, over 200 nm, leading to poor activity in ADR. For instance, Song et al. [22]. reported H_2_ and NH_3_‐fueled PCFCs with peak power density (PPD) of 1017 and 523 mW cm^−2^ at a bare anode under 650°C, respectively. The NH_3_‐PCFC demonstrated a much lower performance than H_2_‐PCFC, with a poor ammonia‐to‐hydrogen PPD ratio (AHR) of 50%. Lin et al. [14]. reported AHR of 65% and 17% for PCFC with Ni‐BZCY anode at 550°C and 450°C, respectively. Additionally, un‐decomposed NH_3_ can react with Ni to form nickel nitride [23], and may lead to the formation of numerous micro‐gaps between BCZYYb grains [24], ultimately degrading cell performance.

**FIGURE 1:**
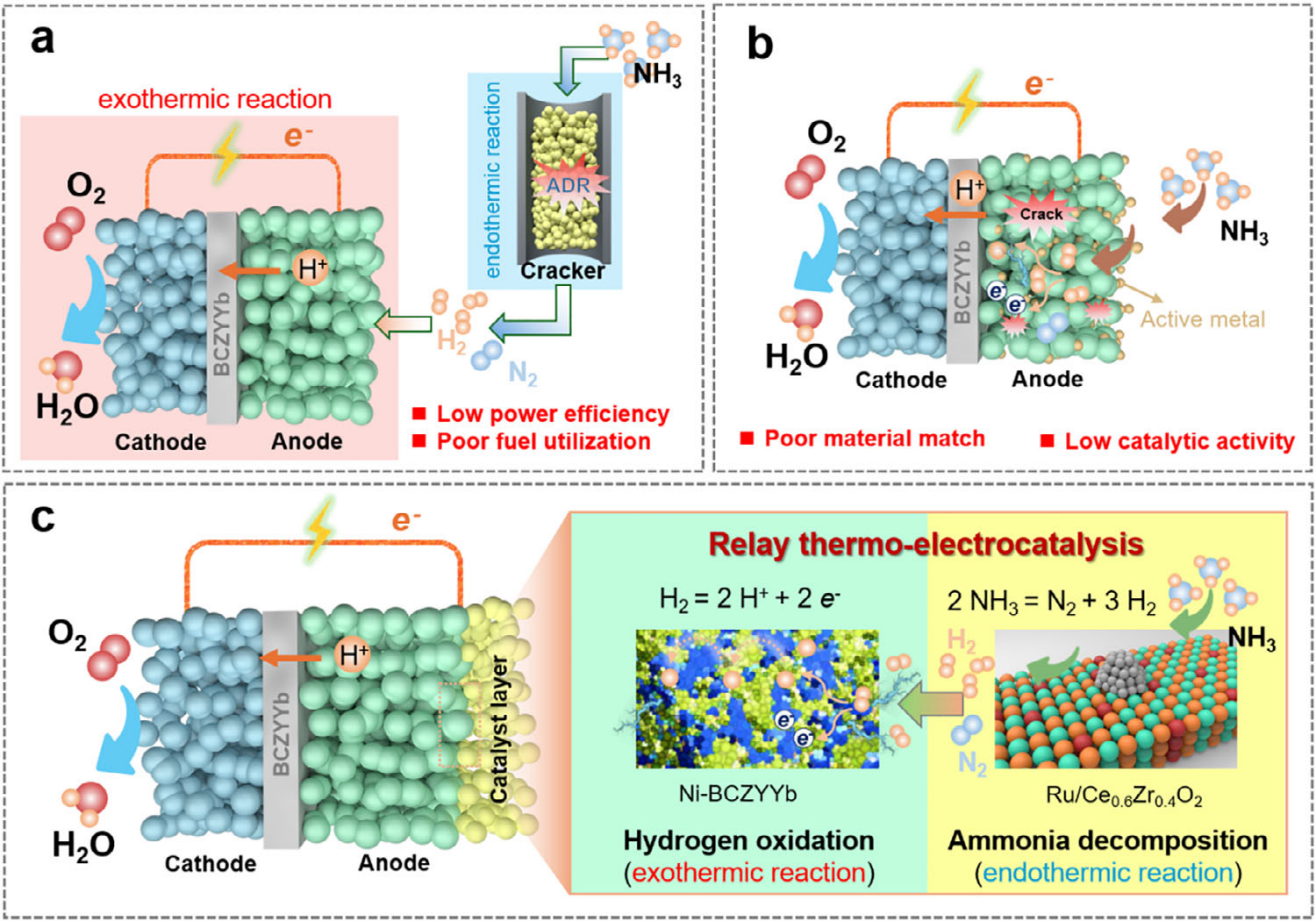
Strategy illustrations for enhanced NH_3_‐PCFCs: (a) External reforming; (b) active metal impregnation; (c) functionalization of catalyst layer.

Motivated by promoting performance by using NH_3_ fuel, various strategies have been employed, as shown in Figure 1. The most common one is by using the external reforming of ammonia for hydrogen production with an ammonia cracker, and then the obtained H_2_ is introduced into the PCFCs for power generation (Figure 1a) [24, 25]. The separated processes reduce the energy efficiency as well as increase the complexity of infrastructures. Instead, decorating anode by impregnating active metal precursors has been demonstrated to have a promotional performance in NH_3_‐PCFCs (Figure 1b). These metal precursors could be reduced and transformed into metal nanoparticles (NPs) for ammonia decomposition. Zhang et al. [17]. dispersed the Fe(NO_3_)_3_ solution on the surface of Ni‐BCZYYb anode, and found that the FeNi_3_ NPs formed on the surface of Ni after reduced by H_2_, thus the PPDs of NH_3_‐PCFCs increased from 1.398 to 1.609 W cm^−2^ at 700°C, which is attributed to the improved ADR. However, these metal particles easily occupy the pores in anodes, resulting in resistance for mass/electron transfer [26]. In addition, the sintering and agglomeration of these NPs induced the expansion of anodes, leading to the degradation and fissuration of the anode layer and electrolyte, leading to the rapid decrease of the cell performance [15, 27].

Herein, we adopt the strategy of catalyst functionalization for enhancing the performance of NH_3_‐PCFCs (Figure 1c), where ammonia undergoes ADR at the catalytic layer and then enters the anode to undergo HOR. This strategy not only enables relay thermo‐electrocatalysis without degrading the energy efficiency, but also does not change the intrinsic structure of the cell [28]. For the efficient ADR of the catalytic layer in NH_3_‐PCFCs, the catalyst needs to be well designed. Currently, ruthenium (Ru) shows the highest ADR activity due to its moderate N─H and N─N bond energies [29]. It can allow complete ammonia conversion at around 500°C when supported on a carrier, making it a promising catalytic layer material for intermediate‐temperature NH_3_‐PCFC. In this work, four supported catalysts, including Ru/BaZr_0.8_Y_0.2_O_3_, Ru/BaCe_0.8_Y_0.2_O_3_, Ru/BaCe_0.8–_ *_x_* Zr*_x_* Y_0.2_O_3_ and Ru/Ce_0.6_Zr_0.4_O_2_ were synthesized using the sol–gel method. Among these, Ru/Ce_0.6_Zr_0.4_O_2_ demonstrated superior ADR, stability, and conductivity. Integrating this catalyst as a layer on the anode significantly improved the AHR of NH_3_‐PCFC. Notably, the performance of H_2_‐PCFC also benefited from the catalyst layer. This research offers valuable insights into the structural design and performance optimization of solid oxide fuel cells using non‐hydrogen fuels.

## 2 Results and Discussion

### 2.1 Relay Thermo‐Electrocatalysis Strategy by ADR Catalyst Functionalization

**FIGURE 2:**
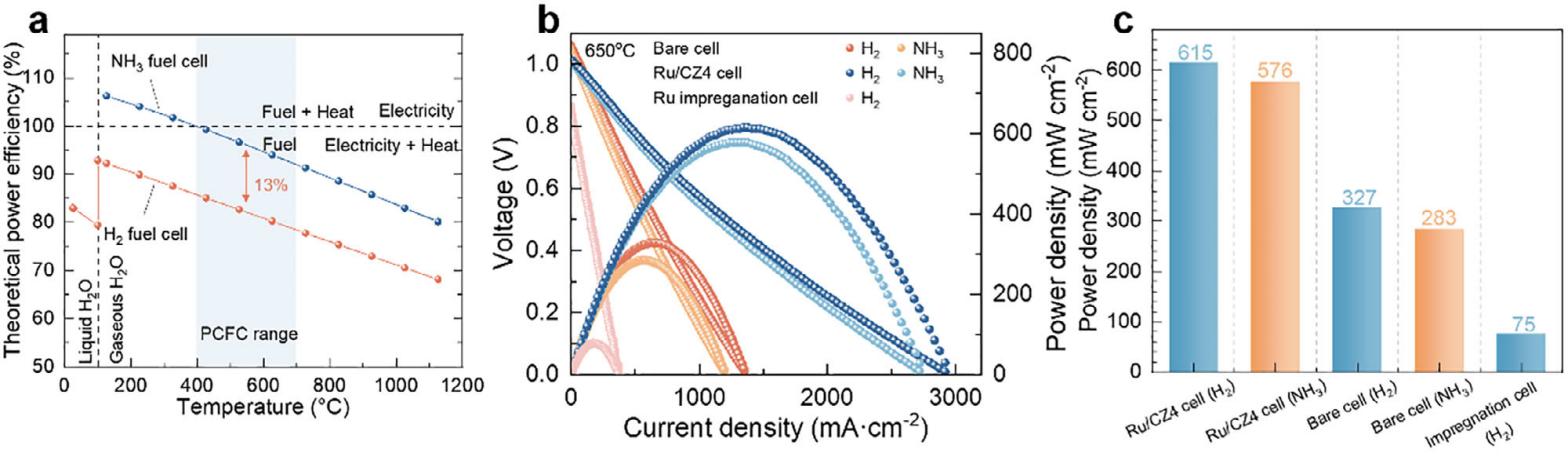
(a) Comparison of theoretical power efficiency of NH_3_ fuel cell and H_2_ fuel cell; (b) *I*–*V*–*P* curves of bare cell, Ru/CZ4 cell, and Ru impregnation cell at 650°C; (c) PPD comparison of bare cell, Ru/CZ4 and impregnation cells by using H_2_ or NH_3_ as fuels.

Figure 2a shows the theoretical power efficiency of fuel cells (*η*_th_) by using H_2_ or NH_3_ at various temperatures, and the definition and the detailed calculation for *η*_th_ can be found in the Supporting Information. The *η*_th_ of NH_3_ fuel cells is ≈92.0%–99.8% within the operational temperature of PCFCs (400°C–700°C), which is 13% higher than that of H_2_ fuel cells. This suggests an efficiency advantage for NH_3_‐PCFCs over H_2_‐PCFCs, which can directly use ammonia as the fuel for power generation. In this work, we adopt the strategy of catalyst functionalization at the anode for NH_3_‐PCFCs. Figure 2b,c compares the performances of the NH_3_‐PCFCs via Ru/Ce_0.6_Zr_0.4_O_2_ (Ru/CZ4) wrapped anode (Ru/CZ4 cell), Ru solution impregnated anode (Ru impregnation cell), and bare cell at 650°C, and the detailed structure and configuration of the cell can be found in Table 1. As shown in the polarization curves (Figure 2b), the Ru/CZ4 cell exhibits the best performance with PPD of 615 and 576 mW cm−^2^ for using H_2_ and NH_3_, respectively, corresponding to an AHR of 93.7%. The bare cell shows a PPD of 327 and 283 mW cm−^2^ for using H_2_ and NH_3_, respectively. It is worth noting that the Ru impregnation cell demonstrates an initial performance with PPD of 75 mW cm−^2^ for using H_2_, and no activity in the case of using NH_3_ as fuel, much lower than other cells. Even though the metal impregnation at anodes is a typical strategy for optimizing the cells, the performance depends on various factors, such as ammonia decomposition activity, phase compatibility, and thermal expansion coefficient between anodes and active components. The poor performance of the Ru impregnation cell might stem from cell deconstruction during the impregnation, reduction, and testing processes. Huang et al. [30] also used an iron catalyst layer, and NH_3_‐PCFCs performance was enhanced by 82% and 32% at 600°C and 650°C, respectively; however, the performance of H_2_‐PCFCs was not provided for comparison. Pan et al. [31] have shown similar results by using an iron‐based catalyst as the catalyst layer. They added Fe‐CeO_2_ catalyst layer inside a tubular NH_3_‐PCFC, which improved the performance by 35.4% and 36% than those of the bare cells at 650°C and 700°C, respectively [5]. Nevertheless, the corresponding AHRs were only 59.2% and 79.7%. That is to say, the performance of NH_3_‐PCFC is still lower than H_2_‐PCFC in the case of catalyst functionalization. He et al. [32]. modified the tubular cell by adding Sr_2_Fe_1.35_Mo_0.45_Cu_0.2_O_6‐δ_ catalyst layer onto the Ni‐BZCYYb anode, and the AHR was only 57.6%. Liang et al. [33]. developed a Pr_0.6_Sr_0.4_(Co_0.2_Fe_0.8_)_0.85_Ru_0.15_O_3‐δ_ anode catalytic layer, where the CoFeRu alloys were exsoluted under a reducing atmosphere; the AHR was 52.5%. Actually, the functionalization of the catalyst layer not only promotes the PPD of the cell but also affects the performance under different fuels. Therefore, the AHR is an important factor to evaluate the adaptability and superiority of the catalyst layers at the anode. Notably, the PPD of the Ru/CZ4 cell using NH_3_ is 104% higher than the bare cell, and the AHR is above 93% in our case, posing the importance of the catalyst functionalization at the anode for promoting the performance of NH_3_‐PCFCs. This indicates that the employment of the Ru‐based catalyst as an anode functionalization layer at 650°C has significantly improved the performance and AHR of NH_3_‐PCFCs.

**TABLE 1:** Material configuration of the PCFC.

Cell type	Modification strategy	Anode	Electrolyte	Cathode
Bare cell	∖	Ni‐BCZYYb	BCZYYb	LSCF‐BCZY
Ru impregnation cell	Ru solution impregnation			
Ru/CZ4 cell	Ru/CZ4 catalytic layer			
Ru/BZY cell	Ru/BZY catalytic layer			
Ru/BCY cell	Ru/BCY catalytic layer			
Ru/BCZY cell	Ru/BCZY catalytic layer			

### 2.2 Importance of the Catalyst Layer for Enhancing the Performance of NH_3_‐PCFCs

**FIGURE 3:**
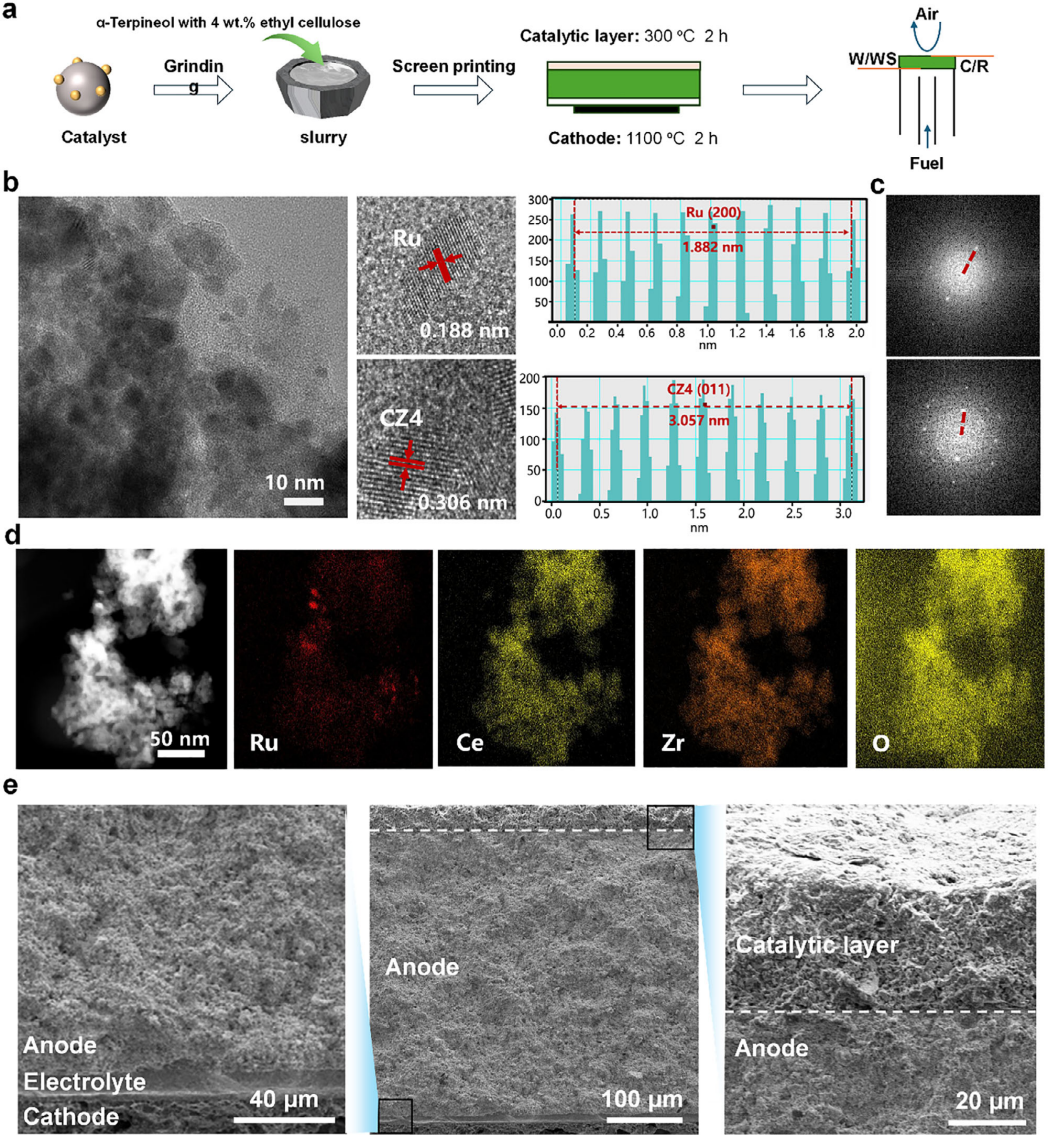
(a) Schematic diagram of the strategy of catalytic layer on NH_3_‐PCFC, (b) HR‐TEM images, (c) fast Fourier transform images, and (d) STEM‐EDX mapping of the Ru/CZ4 catalyst. (e) Cross‐sectional SEM images of the cell after Ru/CZ4 catalyst functionalization.

The catalyst functionalization cells were fabricated by using an integrated process involving the synthesis of catalyst, catalyst slurry, and the screen printing of the anode, as demonstrated in Figure 3a. Initially, the Ce_0.6_Zr_0.4_O_2_ was prepared by a sol–gel method, followed by the deposition of Ru species onto the support, obtaining the Ru/Ce_0.6_Zr_0.4_O_2_ catalyst, see Supporting Information, for details. After precise screening of catalyst particle size, the catalyst slurry was synthesized in the presence of terpineol and ethyl cellulose. The catalyst‐functionalization cell was fabricated by screen printing the ADR catalyst slurry on a raw single cell. The catalyst slurry was printed onto the surface of the anode and baked to form a close contact and compatible anode‐catalyst interface. Figure 3b–e presents the detailed information about the textural structure of Ru/Ce_0.6_Zr_0.4_O_2_. The Ru nanoparticles (NPs) are formed and evenly embedded in Ce_0.6_Zr_0.4_O_2_ support under a reduced atmosphere. The HR‐TEM image (Figure 3b) exhibits clear lattice fringes with interplanar distances of 0.188 nm, corresponding to the (2 0 0) facet of Ru NPs, and a carrier with a crystallographic spacing length of 0.306 nm, corresponding to the (0 1 1) crystallographic plane of Ce_0.6_Zr_0.4_O_2_. Additionally, the fast Fourier transform (FFT) images (Figure 3c) were also analyzed, and the result manifests the (h k l) planes ascribed to the diffractions of Ru species and CZ4. Furthermore, the STEM‐EDX mapping results of Ru/Ce_0.6_Zr_0.4_O_2_ shown in Figure 3d demonstrate that the Ru species are properly distributed in the Ce, Zr, and O domains. These results demonstrate that the Ru NPs were successfully anchored on the surface of Ce_0.6_Zr_0.4_O_2_.

Figure 3e shows the SEM images of the overall cross‐section of the cell with Ru/CZ4 catalyst functionalization. As indicated, the thickness of the cell is around the overall thickness of the anode support PCFC, which was about 350–400 µm. The anode is designed to be thick enough to provide the cell with sufficient mechanical strength. The electrolyte exhibits a dense morphology, indicating that it effectively separates the anode and cathode gases. In contrast, the morphologies of the anode catalyst layer, anode support layer, and cathode layer differ significantly from that of the electrolyte, suggesting the presence of micropores that facilitate gas transport. In our cells, we prepared the anode support layer with a sufficient thickness (≈300 µm) by adjusting the solid loading of the anode powder (NiO‐BZCYYb) slurry because the support requires a certain thickness to support the electrolyte, cathode, and catalyst layer of the PCFCs. The electrolyte layer shows a thickness of 20 µm and is densely sintered. Interestingly, the enlarged image of the black region demonstrated the successful functionalization of the Ru/Ce_0.6_Zr_0.4_O_2_ catalyst onto the anode with a close contact. No cracking was found in the catalyst‐anode interface, indicating good chemical compatibility and a suitable match in thermal expansion behavior between the catalyst and the anode.

**FIGURE 4:**
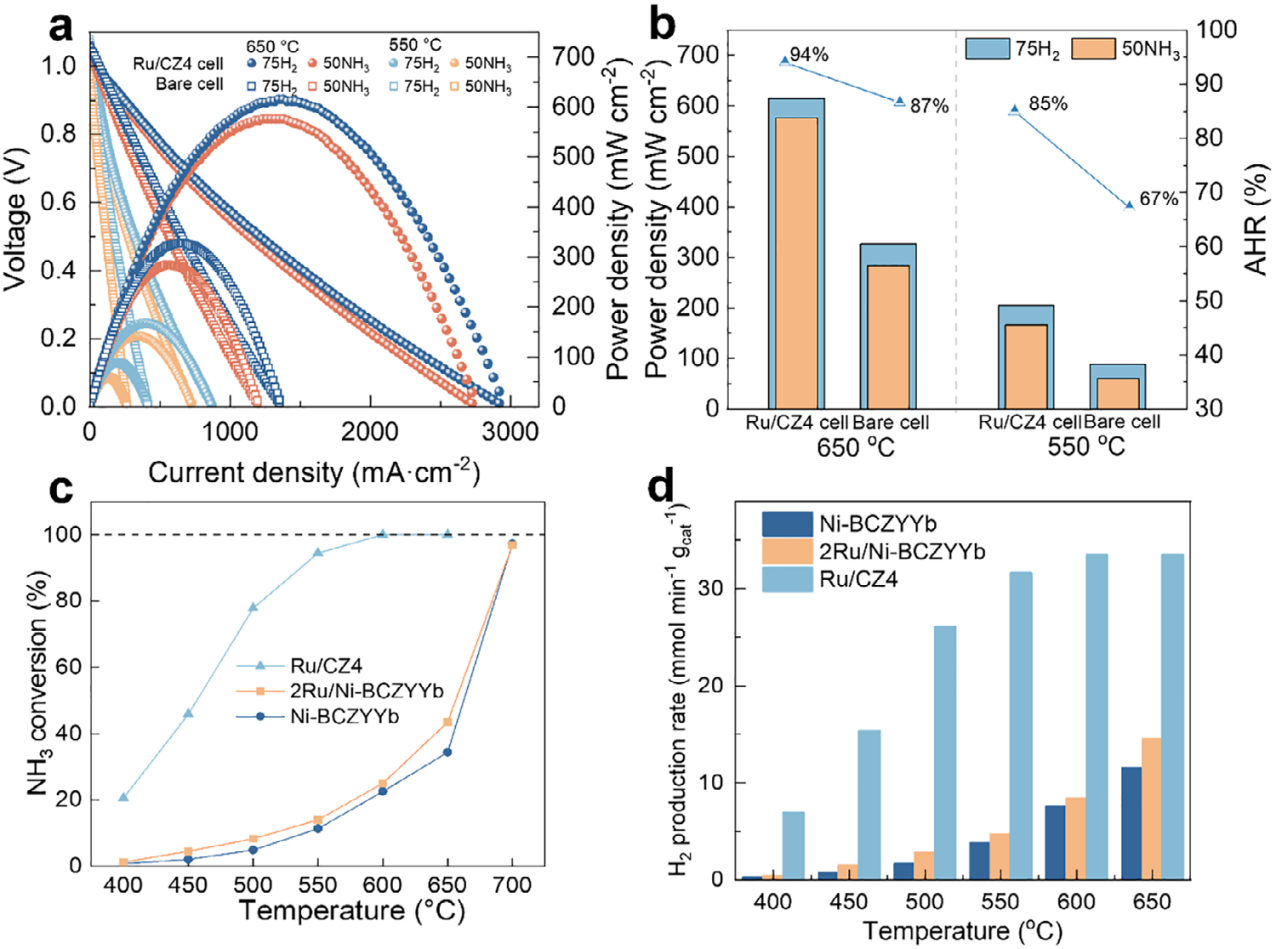
(a) Electrochemical performance and (b) AHR and PPD of the NH_3_‐PCFC with bare cell and Ru/CZ4 cell. (c) Ammonia conversion. (d) H_2_ production rate of Ni‐BCZYYYb, 2Ru/Ni‐BCZYYYb, and Ru/CZ4 catalysts.

To assess the electrochemical performance of the PCFCs (bare cell and catalyst‐functionalization cells), the *I*–*V* and power density curves (Figure 4a) have been measured by using 75 vol%H_2_/25 vol%N_2_ and pure NH_3_ as the fuels at different temperatures, as shown in Figure 4a. In the presence of NH_3_ fuel, the PPD of the PCFC wrapped with Ru/Ce_0.6_Zr_0.4_O_2_ were 576 and 166 mW cm^−2^ under 650°C and 550°C, respectively, which were two‐fold higher than that of the bare cell. It can be seen that the performance of NH_3_‐fueled cell is close to the H_2_‐fueled one in the case of Ru/Ce_0.6_Zr_0.4_O_2_ functionalization, which indicates that the Ru/Ce_0.6_Zr_0.4_O_2_ catalyst enhanced the PCFC performance in the presence of ammonia fuel. The AHR values have a great increase under 550°C and 650°C (Figure 4b), which increase from 67% to 85% and from 87% to 94%, respectively. This indicates that the catalyst‐functionalization strategy has more obvious promotion for performance enhancements of NH_3_‐PCFCs at low temperatures. It is worth mentioning that, besides the performance enhancements, the direct ammonia decomposition at the outersurface of the anode in our work is beneficial for the process intensification because of the fast heat transfer by the closed physical co‐location of the endothermic ammonia decomposition and exothermic electro‐oxidation processes.

To understand the superior performance in the case of catalyst functionalization, the ammonia decomposition was conducted for comparison of different active components. As shown in Figure 4c, the Ni‐BCZYYYb shows poor activity in ammonia decomposition, and the ammonia conversion is only 12% at 550°C. This indicates that the performance of the bare cell is limited by the activity of the supported anode for ammonia decomposition. Interestingly, no enhancement in activity in the Ni‐BCZYYYb incorporated 2 wt% Ru (denoted as 2Ru/Ni‐BCZYYYb), which is used as an anode material in the case of Ru impregnation cell. That is to say, the Ru impregnation did not promote the ammonia decomposition activity. Instead, the Ru/Ce_0.6_Zr_0.4_O_2_ catalyst shows an excellent performance for ammonia decomposition, and the ammonia conversion reached 94% at 550°C and almost complete conversion at 650°C. These results indicate that the Ru/Ce_0.6_Zr_0.4_O_2_ with optimal electrochemical performance has higher ammonia decomposition activity than that of the Ni‐BCZYYYb anode and the Ru‐impregnated anode. Figure 4d summarizes the H_2_ formation rates over the Ru/Ce_0.6_Zr_0.4_O_2_, Ni‐BCZYYYb, and 2Ru/Ni‐BCZYYYb catalysts under different temperatures. The H_2_ formation rate of Ru/Ce_0.6_Zr_0.4_O_2_ is 33.4 mmol g_cat_^−1^ min^−1^ at 650°C, which is superior to that of Ni‐BCZYYYb (11.5 mmol g_cat_^−1^ min^−1^) and 2Ru/Ni‐BCZYYYb (14.5 mmol g_cat_^−1^ min^−1^). This is consistent with the electrochemical performance results. The functionalization of the Ru/Ce_0.6_Zr_0.4_O_2_ catalyst enables the PCFC to perform an excellent activity by using pure ammonia as the fuel under lower temperatures.

### 2.3 Design of Well‐Matched ADR Catalysts and General Principle

**FIGURE 5:**
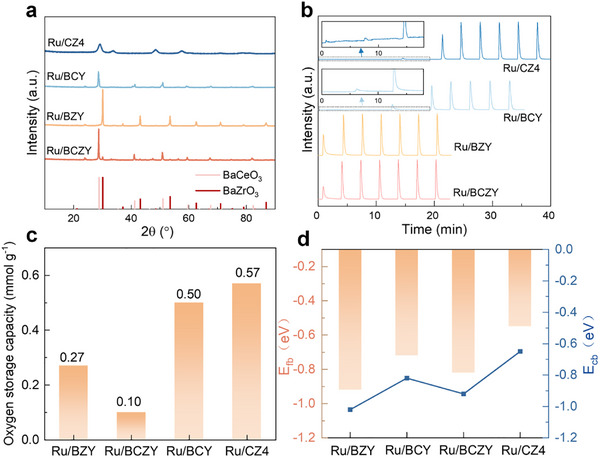
(a) XRD profiles, (b) O_2_ pulse chemisorption spectrum, (c) oxygen storage capacity, and (d) surface voltage of the Ru/BZY, Ru/BCZY, Ru/BZY, and Ru/CZ4 catalysts.

As discussed above, the catalysts for efficient ammonia decomposition are significantly important for improving the performance of NH_3_‐PCFCs. Motivated by the design of well‐matched ADR catalysts, a series of catalysts was prepared for evaluation and understanding the structure–performance relationship. As shown from the X‐ray diffraction (XRD) patterns (Figure S1, Supporting Information), a single cubic fluorite structure was found in the Ce_0.6_Zr_0.4_O_2_ (PDF#38‐1436). In contrast, the BaZr_0.8_Y_0.2_O_3_ (BZY), BaCe_0.8_Y_0.2_O_3_ (BCY), and BaCe_0.8−_ *_x_* Zr*_x_* Y_0.2_O_3_ (BCZY) displayed a cubic perovskite structure according to the XRD analysis. It can be seen that the XRD patterns show no obvious change after Ru loading with H_2_ reduction (Figure 5a). No diffraction peak of Ru species was found, which indicates the well dispersion of Ru on these supports. The typical morphologies and structures of these catalysts are shown in Figures S2 and S3, Supporting Information. Furthermore, the XPS analysis was performed to investigate the surface element compositions, chemical states, and electronic properties of these samples. Figure S4, Supporting Information, shows the XPS survey spectra of Ru/BCY, Ru/BZY, Ru/BCZY, and Ru/CZ4 samples. The Ce 3d spectra in Ru/BZY, Ru/BCZY, and Ru/BCY are presented in Figure S5, which could be deconvoluted into ten peaks by the Gauss–Lorentz model function. The surface content of Ce^4+^ and Ce^3+^ was analyzed, and the results show that the Ce^3+^ content accounted for 41.8, 45.2, and 34.7 mol% of the total Ce contents (Ce^4+^ +Ce^3+^) in Ru/BCZY, Ru/BCY, and Ru/CZ4, respectively. The XPS profiles of Zr 3d in Ru/BZY, Ru/BCZY, and Ru/CZ4 are illustrated in Figure S6, Supporting Information. The B‐sites in Ru/BZY are dominated by Zr species (80 mol%), while the B‐sites in Ru/BCZY have only 10 mol% Zr. In the case of Ru/BZY, some Zr^4+^ was reduced to sub‐oxide Zr. These species accounted for 51.0 mol% of the total Zr content, which is less than that of the Zr in Ru/BCZY (80.4 mol%). That is to say, the incorporation of Zr in BCY prevents the reduction of Ce^4+^ to Ce^3+^. The generation of abundant sub‐oxide Zr species further promotes the formation of oxygen vacancies and enhances the stabilization of Ce species in the lattice.

As is well known, the oxygen vacancy plays a crucial role in electrode materials for ammonia decomposition and solid oxide fuel cells, which is beneficial to facilitating electron and ion transfer through the fuel cells [34, 35]. Initially, the XPS profiles of the O 1s were analyzed to identify the presence of oxygen vacancies (Figures S7 and S8, Supporting Information). It is generally believed that the content of oxygen vacancies can be determined by deconvolution of O peaks. According to the literature [36], the presence of O vacancies (O_v_) makes the adsorption of surficial active oxygen on the catalyst surface, generating the absorbed O species (O_ads_). The high proportion of O_ads_ implies a higher O_v_ concentration. Thus, the concentration of O_ads_ and O_v_ proportion would reflect the amount of oxygen vacancies of the catalysts. The results identify the presence of oxygen vacancies in all the catalysts, which is further confirmed by the EPR spectra (Figure S9, Supporting Information). Nevertheless, due to the inherent differences in oxygen vacancy content between different oxide structures, direct comparisons based solely on XPS data might not accurately reflect the effective oxygen vacancy concentration. To address this issue, we conducted O_2_ pulse chemisorption tests to assess the effective oxygen adsorption capacity of each catalyst, with the spectrum and the oxygen storage capacity (OSC) of different catalysts presented in Figure 5b,c, respectively. The OSC results indicate that Ru/CZ4 has the highest OSC (0.568 mmol g^−1^), followed by Ru/BCY (0.497 mmol g^−1^) and Ru/BZY (0.269 mmol g^−1^), while Ru/BCZY exhibits the lowest (0.102 mmol g^−1^). These results suggest that Ru/CZ4 possesses a greater number of effective oxygen vacancies than Ru/BZY, Ru/BCZY, and Ru/BCY. It is generally believed that the O vacancy is one of the key factors to evaluate the electronic property, ion/electron transfer ability, and interaction between the support and the metal species, involving the concentration of the O vacancy and the O^2−^ migration rate. Therefore, the regulation of oxygen vacancy concentration is important toward superior electrocatalytic activity and higher ionic conductivity in NH_3_‐PCFCs.

Besides, the chemical states of Ru species were also investigated over the Ru/BZY, Ru/BCZY, and Ru/BZY catalysts, as demonstrated in Figure S10, Supporting Information. The Ru 3p profiles were analyzed based on the calibration of the C 1s standard peak (284.8 eV). The typical peaks over these samples show two distinct asymmetric peaks, indicating the presence of two Ru species on the catalyst surface. The peak located at the low binding energy region corresponds to Ru^0^ species, and that in the high binding energy region is attributed to Ru*^n^* ^+^ species. For perovskite, the presence of oxygen vacancies and the low‐valent metal ions facilitates electron transfer between the support and the Ru*^n^* ^+^, which leads to the formation of electron‐rich Ru and promotes the generation of Ru^0^. As the increase in surface oxygen vacancies, the electron density of the surface Ru species would increase, allowing Ru*^n^* ^+^ to gain electrons and convert into active Ru^0^. As a result, the amount of Ru^0^ typically increases with the concentration of oxygen vacancies. The Ru/BCZY shows a higher content of Ru^0^ than Ru/BCY and Ru/BZY, which consists with the results of O 1s profiles. It can be seen that the percentage of Ru^0^ species in Ru/CZ4 is much higher than in other samples, which is presumably due to the higher OSC of CZ4, and then facilitating the enrichment of electrons on Ru under reduction atmosphere. These results suggest that increasing oxygen vacancies allows the Ru sites to perform better activity for ammonia decomposition, which is presumably due to the donation of electrons of oxygen vacancies, facilitating the reduction of Ru*^n^* ^+^ to Ru^0^ and thereby enhancing ammonia decomposition activity. To better understand the band structures, the Mott‐Schottky curves were further conducted over the Ru/BZY, Ru/BCZY, Ru/BCY and Ru/CZ4 (Figure S11, Supporting Information). The Mott–Schottky curves were measured at three frequencies of 500, 1000, and 1500 Hz, utilizing a Pt electrode as the counter electrode and an Ag/AgCl electrode as the reference. The voltage of the flat band (*E*_FB_) and conduction band (*E*_CB_) was calculated and analyzed, as shown in Figure 5d. The *E*_CB_ are found to be −1.02, −0.92, −0.82, and −0.65 eV, respectively. The negative conduction band is feasible for electrochemical cycling reaction via electron transfer.

### 2.4 Performance

**FIGURE 6:**
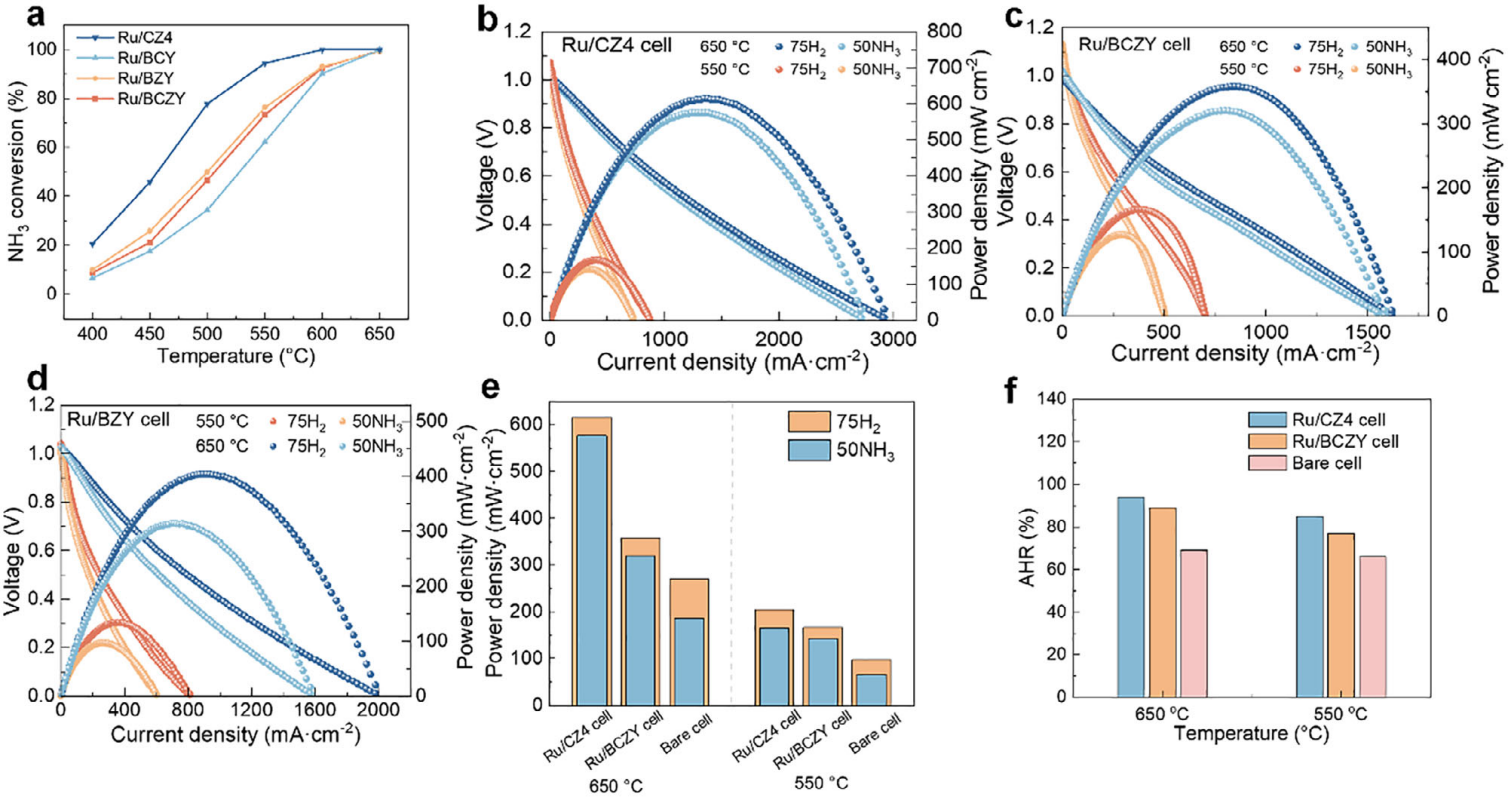
(a) Ammonia conversion over Ru/BZY, Ru/BCZY, Ru/BZY, and Ru/CZ4 catalysts. (b–d) *I*–*V*–*P* curves of Ru/BZY cell, Ru/BCZY cell, and Ru/CZ4 cell. (e) PPD and (f) AHR of Ru/BZY cell, Ru/BCZY cell, and Ru/CZ4 cell.

To evaluate the catalytic ability in NH_3_‐PCFCs over the Ru/BZY, Ru/BCZY, Ru/BCY, and Ru/CZ4 catalysts, the ammonia decomposition was conducted, and the results are demonstrated in Figure 6a. As expected, the ammonia conversion increases with the increase in temperature over all samples, which reveals the enhanced cell performance under elevated temperatures. It can be seen that the Ru/CZ4 displays a much higher ammonia conversion than those of Ru/BZY, Ru/BCZY, Ru/BZY, indicating the superior hydrogen production when it is employed in an integrated catalyst‐anode surface. The Ru/CZ4 cell, the cell with Ru/BZY wrapped anode (Ru/BZY cell), and the cell with Ru/BCZY wrapped anode (Ru/BCZY cell) were fabricated, and the detailed configuration of the cell can also be found in Table 1. We tested these cells for comparison of the power density in the cases of catalyst functionalization under 75 vol%H_2_/25 vol%N_2_ and pure NH_3_ gas. The results were shown in Figure 6b–d. The Ru/BZY cell exhibits a satisfying electrochemical performance with 404 mW cm^−2^ at 650°C and 134 mW cm^−2^ at 550°C under 75 vol%H_2_/25 vol%N_2_. However, the performance under NH_3_ decreases obviously, with only 315 mW cm^−2^ at 650°C and 97 mW cm^−2^ at 550°C, respectively. The PPD value of the Ru/BCZY cell is 358 mW cm^−2^ at 650°C and 166 mW cm^−2^ at 550°C in under 75 vol%H_2_/25 vol%N_2_ and 320 mW cm^−2^ at 650°C and 127 mW cm^−2^ at 550°C in NH_3_. For comparison, the bare cell was also evaluated (Figure S12, Supporting Information). The PPD and the AHR in both cases of 75 vol%H_2_/25 vol%N_2_ and NH_3_ are much lower than those of the catalyst functionalization ones. It can be seen that the Ru/CZ4 cell achieves the highest PPD value of 615 mW cm^−2^ at 650°C and 167 mW cm^−2^ at 550°C under 75 vol%H_2_/25 vol%N_2_. When the fuel is switched to NH_3_, the cell exhibits PPD values of 576 mW cm^−2^ at 650°C and 142 mW cm^−2^ at 550°C. Figure 6e compares the PPD values in the cases of different cells, fuel gases, and temperatures. For a better understanding of the effect of catalyst functionalization on the performance enhancement, the AHR was further calculated as shown in Figure 6f. The AHR of Ru/CZ4 cell is 85% and 94%, respectively, which is 22% and 25% higher than those of the bare cell (66% and 69% at 550°C and 650°C). The low AHR is presumably due to the insufficient catalytic ammonia decomposition over the Ru/BZY and Ru/BCZY, even though they have lower *E*_FB_ and *E*_CB_. These results reveal that the catalyst functionalization not only promotes the performance of NH_3_‐PCFCs (in the case of H_2_ and NH_3_) but also enhances the absolute performance in the same fuel gas compared with bare cells.

In addition, the Nyquist plots were demonstrated for a better understanding of the performance differences (Figure S13, Supporting Information). Generally, the intercept of the curve with the real axis on the high‐frequency side (left side) and the low‐frequency side (right side) represents the cell's ohmic resistance (*R*_o_) and the total impedance (*R*_total_), respectively, and the polarization resistance is *R*_total _− *R*_o_ (*R*_P_). The *R*_p_ is larger than *R*_o_ for the bare cell at all operating temperatures, suggesting the significant contribution of *R*_p_ to the total resistance in PCFCs. The smaller *R*_o_ values are insensitive to operating temperatures and gas atmosphere, attributed to the low ohmic resistance of thin electrolyte layers in the cases of anode support cells. It can be seen that *R*_p_ increases rapidly as the decrease of the operating temperatures, which is due to the low diffusion coefficient of ammonia and the sluggish ammonia oxidation reaction process, and then limits the mass transfer of gas. The functionalization of catalysts on the anode surface allows the ammonia to decompose into H_2_ and N_2_ first at the anode. This increases the diffusion of H_2_ and then decreases the *R*_p_. Compared with bare cells, the catalyst‐functionalization cells show much lower *R*_p_ values, as illustrated in Figure S13, Supporting Information. The impedances of Ru/CZ4 cell are almost the same in the cases of 75 vol%H_2_/25 vol%N_2_ and pure NH_3_ at 650°C, which is consistent with the above performance results. The *R*_p_ value is only half of the bare cell, which indicates that the Ru/CZ4 incorporation can accelerate the electrochemical reaction greatly. Compared with the bare cell, the high ammonia decomposition activity of Ru/CZ4 at low temperatures and the rapid transfer of hydrogen species generated from ammonia will undoubtedly improve the overall performance of the NH_3_‐PCFCs. To assess the effect of the catalytic layer on the stability of NH3‐PCFC, we tested the Ru/CZ4 cell under an ammonia atmosphere at 0.6 V, using the bare cell as a control. As shown in Figure S14, Supporting Information, the bare cell exhibited a significant decline in current density after 10 h, while the Ru/CZ4 cell maintained a stable current density between 180 and 200 mA cm^−2^ throughout the test. Additionally, the stability curve of the bare cell showed substantial fluctuations, whereas the Ru/CZ4 cell's curve remained steady. These results indicate that the Ru/CZ4 catalytic layer significantly improves the stability of NH_3_‐PCFC.

### 2.5 Insight Into the Incorporated Anode for Superior Performance of NH_3_‐SOFCs

**FIGURE 7:**
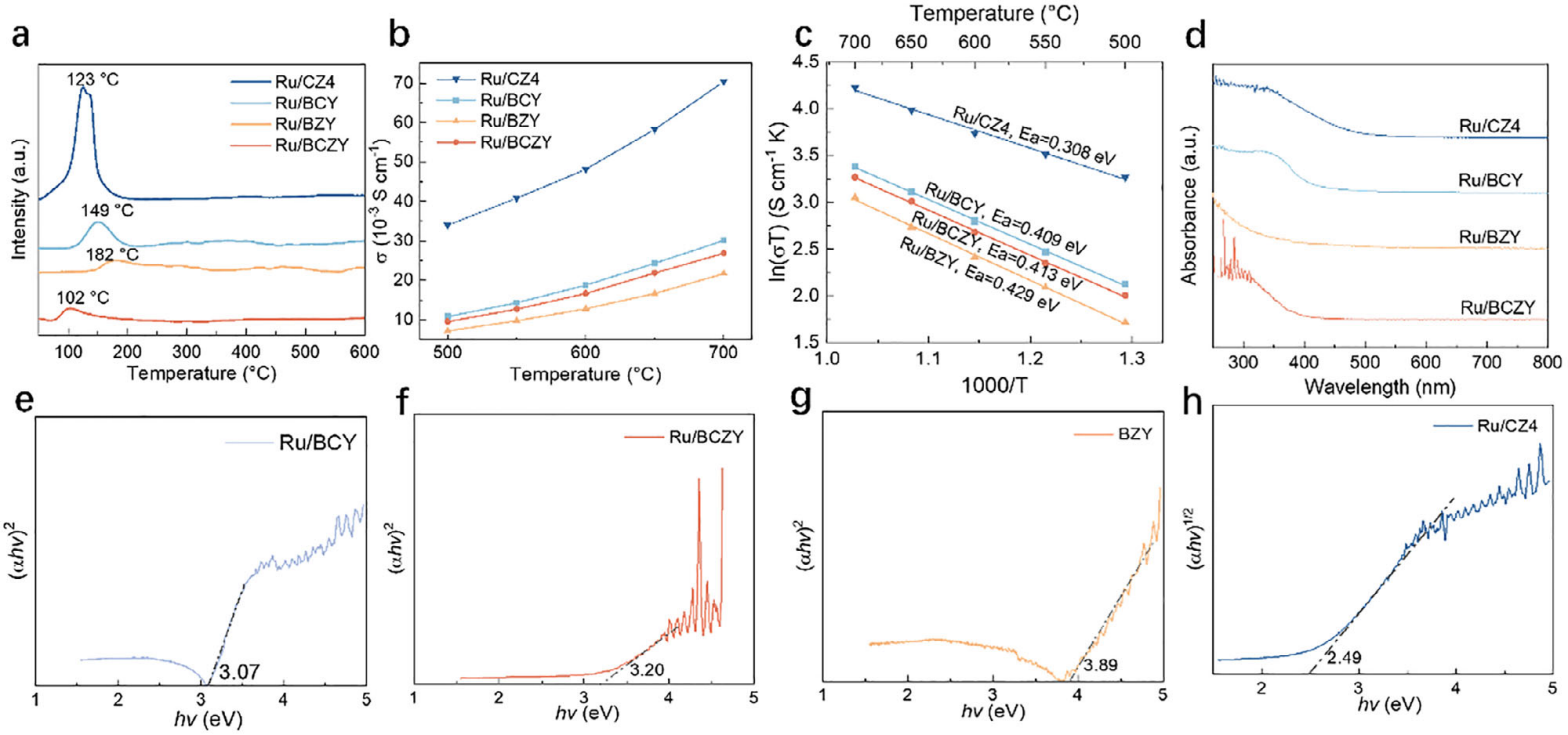
(a) H_2_‐TPD profiles, (b) electrical conductivities, (c) Arrhenius plots of electrical conductivities, (d) optical absorption spectra, and (e–h) Tauc curves of the Ru/BCY, Ru/BCZY, Ru/BZY, and Ru/CZY.

To gain insight into the fundamental understanding of the superior performance of NH_3_‐SOFCs with catalyst functionalization, a series of characterizations was conducted. The H_2_‐TPD profiles were conducted at a temperature range of 50°C–600°C to investigate the adsorption and desorption properties of hydrogen species on Ru/BZY, Ru/BCZY, Ru/BCY, and Ru/CZ4 catalysts (Figure 7a). Notably, all samples exhibited an H_2_ desorption peak below 200°C, attributed to the desorption of hydrogen species on the catalyst surface. As is well known, the Ru surface always suffers from hydrogen poisoning due to the strong interaction between H_2_ and Ru species, where the reaction active sites would be blocked, leading to a decrease in the catalytic activity. The hydrogen poisoning diminishes the amount of NH_3_ adsorbed on the active sites, which reduces the ammonia conversion. Among these samples, the Ru/CZ4 demonstrated a low H_2_ desorption temperature of 123°C with a large peak of hydrogen desorption. This indicates the Ru/CZ4 possesses the superior ability of hydrogen activation and desorption, which is beneficial for the ammonia decomposition and the hydrogen hopping on the surface. In contrast, the Ru/BCY and Ru/BZY demonstrated higher temperatures and much smaller peaks for hydrogen desorption. Even though the desorption peak of hydrogen of the Ru/BCZY is around 102°C, the peak is small, which manifests the poor activation ability of hydrogen. These findings suggest that the Ru/CZ4 is more effective in transferring hydrogen species from the Ru active site to the carrier, thereby mitigating hydrogen poisoning more effectively.

**FIGURE 8:**
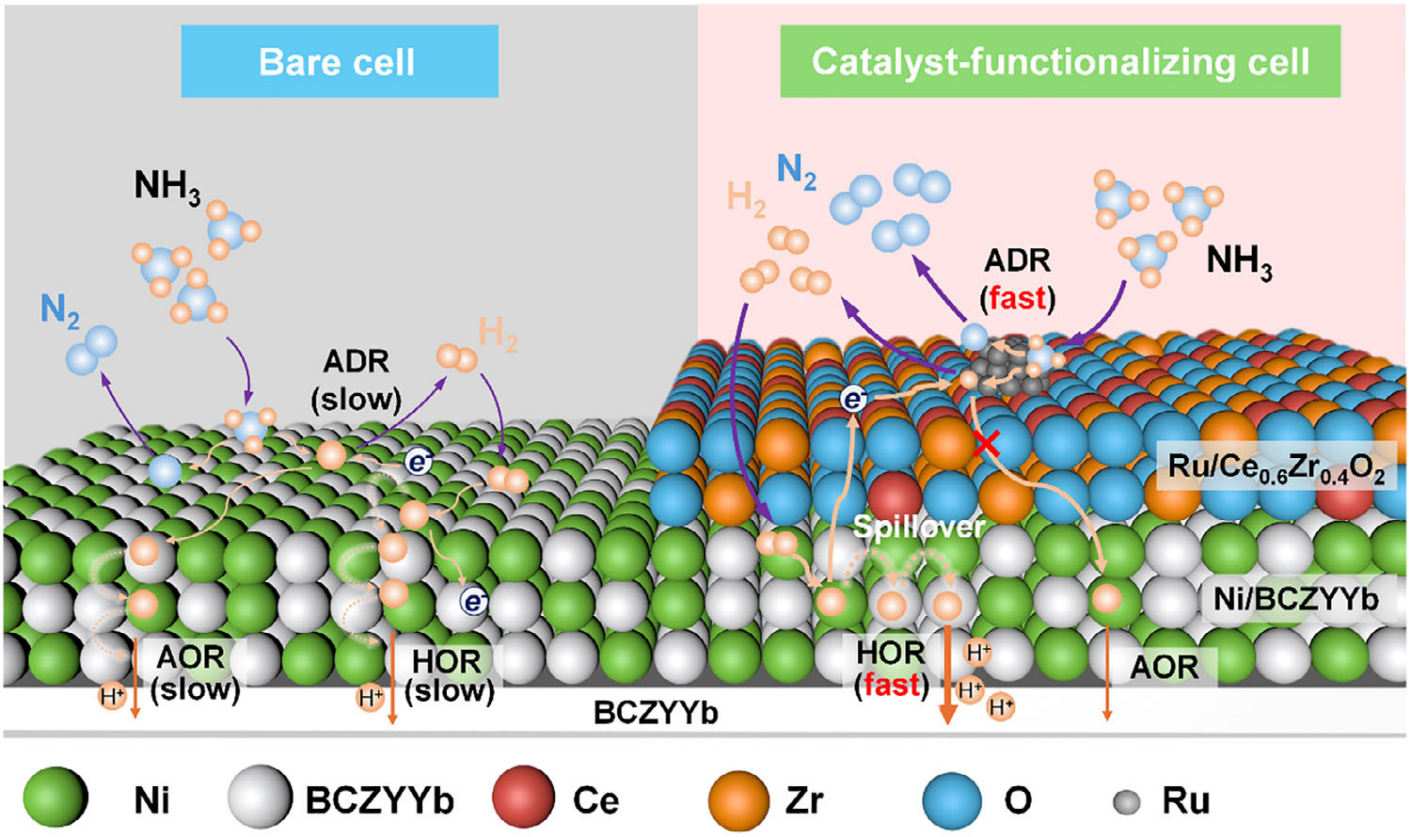
Schematic of the mechanism of the bare cell and catalyst‐functionalization cell.

In order to explore the conductivity of the catalysts, the DC four‐electrode method was used to test the conductivity at 500–700°C in a dry H_2_ atmosphere. As illustrated in Figure 7b, the conductivity of all samples increases with the increase of the temperatures, aligning with the conductive behavior of n‐type semiconductors. This is due to the narrow band gap in the semiconductors with the increase of temperatures, enabling more valence electrons to break covalent bonds and transition to the conduction band and thereby generating a higher concentration of flowing charges. Consequently, a higher carrier concentration leads to lower resistivity for the semiconductor. The conductivity increases more rapidly at higher temperatures due to the significant escape of lattice oxygen from the carrier structure. While excessive oxygen vacancies may hinder electron conduction, they also facilitate the absorption of more protons, accelerating proton conduction, and enhancing material conductivity. The conductivity order is as follows: Ru/BZY < Ru/BCZY < Ru/BCY < Ru/CZ4. According to the Arrhenius plots, the conductivity activation energies of the samples were calculated, and the results are shown in Figure 7c. It is found that ln(*σT*) has a good linear relationship with 1000/*T*, indicating that the ion conduction of the proton conductor is a thermal activation process. Specifically, the conductivity activation energies for Ru/BZY, Ru/BCZY, Ru/BCY, and Ru/CZ4 are 0.429, 0.413, 0.409, and 0.308 eV, respectively. During the B‐site transition from Zr^4+^ to Ce^4+^, the conductivity activation energy gradually decreased. Therefore, Ru/BCZY and Ru/BCY exhibit lower conductivity activation energies and lower energy barriers for proton transport, resulting in higher conductivity compared to Ru/BZY. For Ru/CZ4, the gradual increase in doping with small ion radius Zr^4+^ leads to an increase in oxygen vacancies, which drives the conversion of Ce^4+^ to Ce^3+^. The continuous generation of Ce^3+^ further induces the formation of oxygen vacancies. Therefore, Ru/CZ4 has the lowest conductivity activation energy and exhibits the highest conductivity within the cell's operating temperature range.

The UV–vis diffuse reflectance spectra were employed to analyze the band structures of these samples within the 250–800 nm range (Figure 7d–g). The energy difference between the band's bottom and the valence band's top is termed the band gap width, also referred to as the band gap. The band gap width (denoted as *E*_g_) essentially reflects the binding strength of the valence band electrons, that is, the minimum activation energy required for the electrons to transition from the valence band to the conduction band. A smaller *E*_g_ value translates to a lower energy threshold for electron excitation, thereby enhancing the material's conductivity. In our cases, the absorption peak can be observed in the 300–400 nm region, which is mainly due to the excitation transition of the charge from the valence band to the conduction band (Figure 7d). The *E*_g_ values of the catalyst can be estimated by extending the tangent of the UV–vis spectrum to the *X*‐axis: the band gap widths of Ru/BZY, Ru/BCZY, Ru/BCY, and Ru/CZ4 are about 3.89, 3.20, 3.07, and 2.49 eV, respectively. Ru/BZY boasts the highest *E*_g_ value, indicating a broader energy gap between the conduction and valence bands, and a correspondingly higher energy requirement for electron excitation, resulting in relatively poor conductivity. Conversely, Ru/CZ4 possesses the lowest *E*_g_ value and demonstrates superior conductivity, which aligns with the conductivity test results.

Based on the above discussion, the preferential reaction mechanism was proposed as demonstrated in Figure 8. On the anode surface of a bare cell, the ammonia decomposes first into H_2_ and N_2_, followed by the hydrogen adsorption and the hydrogen oxidation for power generation at the three‐phase boundary of the anode. In the meantime, the direct ammonia oxidation is presumably to occur on the surface to generate N_2_ and protons. The complex and sluggish processes result in inefficiency for power generation at the Ni‐BCZYYb anode, which shows poor activity for ammonia decomposition. By contrast, the Ru/CZ4 displays an excellent activity for ammonia decomposition. In the case of the Ru/CZ4 cell, the ammonia decomposes initially at the Ru/CZ4 layer to produce H_2_ and N_2_. The H_2_ can be transported to the Ni‐BCZYYb anode due to the excellent ability of hydrogen activation and desorption for proton hopping. Then the hydrogen is followed by adsorption and oxidation at the three‐phase boundary. The incorporation of the Ru/CZ4 catalyst layer separated the ammonia decomposition/oxidation and hydrogen oxidation on different active sites with interoperation that can achieve high power density in NH_3_‐PCFCs. Figure S15, Supporting Information, summarizes the PPDs of NH_3_‐PCFC with catalyst layers affected by electrical conductivities and ammonia decomposition activity. Clearly, the high ammonia decomposition activity and high electrical conductivity of the catalytic layer both contribute to the enhanced performance of the NH_3_‐PCFC. Our results offer valuable insights into the catalyst design and performance optimization of solid oxide fuel cells using hydrogen‐rich fuels.

## 3 Conclusion

In this work, a strategy of ammonia decomposition catalyst functionalization was employed to modify the anode in NH_3_‐PCFCs. Compared to bare cells, the PPD of Ru/CZ4 cell achieves 615 and 576 mW cm−^2^ for using H_2_ and NH_3_, respectively, which are 1.8‐ and 2.0‐fold higher than those of bare cells (327 and 283 mW cm−^2^ for using H_2_ and NH_3_). Importantly, the ammonia‐to‐hydrogen PPD ratio reaches to 93.7%, revealing the superior performance in both hydrogen and ammonia fuels. A series of Ru‐based catalysts (Ru/BZY, Ru/BCZY, Ru/BCY, and Ru/CZ4) was synthesized, evaluated, and characterized for the design of well‐matched ADR catalysts in NH_3_‐PCFCs. The results demonstrated that the activity of ammonia decomposition is the key factor, as well as the electronic properties of these catalysts, for enhancing the performance of NH_3_‐PCFCs. In addition, the detailed structure‐performance relationship and enhancement mechanisms were further investigated and discussed. A balance of high‐performance ammonia decomposition and electrical conductivity of functionalization catalysts was achieved by designing fluorite‐structured catalysts, which are well‐matched to the anode of PCFCs. In summary, the proposed relay thermo‐electrocatalysis strategy by Ru/CZ4 functionalization, integrating the advantages of high ionic conduction, and good electrode catalytic function, is capable of enhancing the reaction kinetics and delivering high performance in NH_3_‐PCFCs.

## Author Contributions

**Huihuang Fang**: experiments, writing – review and editing, supervision. **Zefeng Wang**: experiments, data analysis. **Jiangping Chen**: writing, data analysis. **Jiacheng You**: writing, data analysis. **Yiting Jiang**: writing. **Puxin Yang**: experiments, data analysis. **Qian Lin**: experiments, data analysis. **Haoyu Zhang**: experiments, data analysis. **Fulan Zhong**: writing, data analysis. **Yu Luo**: writing – review and editing, supervision. **Lilong Jiang**: funding acquisition, project administration, supervision.

## Conflicts of Interest

The authors declare no conflicts of interest.

## Supporting information




**Supporting File 1**: exp270125‐sup‐0001‐SuppMat.docx.

## Data Availability

The original data are available from the corresponding authors upon reasonable request.
