# Supplementary material for: Catalyst Functionalization for Elevating Performance in Ammonia Protonic Ceramic Fuel Cells via Relay Thermo‐Electrocatalysis
Source: Exploration (Beijing). 2026 Feb 10;6(1):20240431. doi: 10.1002/EXP.20240431 (PMC12970153; doi:10.1002/EXP.20240431)
Supplement: Supplementary file 1 — Supporting File 1: exp270125‐sup‐0001‐SuppMat.docx. [file EXP2-6-20240431-s001.docx]

**Supporting Information**

**Catalyst functionalization for elevating performance in ammonia protonic ceramic fuel cells via relay thermo-electrocatalysis**

Huihuang Fang,^a,b^ Zefeng Wang,^a^ Jiangping Chen,^a^ Jiacheng You,^a^ Yiting Jiang,^a,b^ Puxin Yang,^a^ Qian Lin,^a^ Haoyu Zhang,^a^ Fulan Zhong,^a,b^ Yu Luo,^a,b*^ Lilong Jiang^a,b*^

^a^ National Engineering Research Center of Chemical Fertilizer Catalyst (NERC-CFC), School of Chemical Engineering, Fuzhou University, Fujian, 350002, China

^b^ Qingyuan Innovation Laboratory, Quanzhou, Fujian 362801, China

^*^ Corresponding author: Yu Luo, [luoy@fzu.edu.cn](mailto:franklin@fjirsm.ac.cn); Lilong Jiang, jll@fzu.edu.cn

1. **Experimental**

**1.1 Preparation of cells**

**1.1.1 Fabrication of the anode support**

BCZY–NiO–cornstarch–PVB (5:5:2.5:0.125) were weighed, mixed, and grounded in proportion to prepare the anode powder. A mixture of 0.6 g/0.5 g was pressed into a pellet and maintained at 15 MPa for 3 minutes. After removal from the mold, the sample was placed in a muffle furnace and heated at a rate of 1 °C/min to 600 °C, held for 1 hour to burn off organic components (with slow heating), and then further heated at a rate of 2 °C/min to 1000 °C, where it was maintained for 2 hours to obtain the anode support.

**1.1.2 Preparation of the full cell**

After the anode support is cooled to room temperature, it is placed on a vacuum spin coater and coated with a layer of NiO–BCZY electrolyte slurry. The coated support is then placed in a muffle furnace, heated at a rate of 2 °C/min to 80 ℃, and held for 1 hour, followed by heating at 2 ℃/min to 600 ℃ and holding for 2 hours. Once cooled to room temperature, the half-cell is removed and a BCZY electrolyte layer is subsequently dip-coated. After coating, the sample is placed in a muffle furnace, heated at 2 °C/min to 80 °C and held for 1 hour, then further heated at 2 °C/min to 600 °C, followed by heating at 1°C/min to 1150/1250/1350/1450 °C and held for 2 hours. After cooling to room temperature, the half-cell is obtained.The LSCF cathode slurry is then applied to the other side of the BCZY electrolyte using a screen-printing method. After coating, the sample is placed in a muffle furnace, heated at a rate of 2 °C/min to 1100 °C, and held for 2 hours. Finally, the NiO–BCZY/BCZY/LSCF–BCZY anode-supported single-cell is obtained.

**1.2 Preparation of Ru/Ce_1–x_Zr_x_O_2_ catalytic layer**

**Preparation of** **Ce_0.6_Zr_0.4_O_2_ supports.** The Ce_0.6_Zr_0.4_O_2_ (CZ4) supports were prepared through a sol–gel method. The raw materials Ce(NO_3_)_3_·6H_2_O (Macklin, AR) and Zr(NO_3_)_4_·5H_2_O (Macklin, AR) were stoichiometrically weighed and dissolved in deionized water, and the citric acid (CA, Macklin, AR) was added into the well–mixed salt solution to facilitate the complexation process. The molar ratio of metal ions to citric acid was 1:1.5. Subsequently, the mixed solution was continuously stirred at 90 °C with water bath to get the gel–like substance. Then the obtained gel was dried in an oven at 180 °C for 24 h to get the precursor powder. Finally, the powder was calcined in a muffle furnace at 800 °C for 5 h to obtain the CZ4 supports.

**Preparation of Ru/Ce_0.6_Zr_0.4_O_2_ catalysts.** Ru/CZ4 catalysts were prepared through an impregnation method. Then a certain amount of Ru(NO)(NO_3_)_3_ solution (Ru(NO)(NO_3_)_x_(OH)_y_, x+y=3, Macklin, Ru 1.5% w/v) was added to the CZ4 supports drop by drop to yield a 2 wt.% Ru loading. After that, the precursor samples were dried under an incandescent lamp, followed by sintering at 300 °C for 2 h to get the Ru/CZ4 catalysts.

**Preparation of** **cells with catalyst functionalization**. Nickel oxide and prepared Ru/CZ4 catalyst were finely grounded and mixed in a mass ratio of 6:4. Then, a certain amount of 4 wt% ethyl cellulose (C_23_H_24_N_6_O_4_) doped terpineol (C_10_H_18_O) was added into the mixed nickel oxide and Ru/CZ4 powder to get the anode catalytic layer slurry. The catalytic slurry was coated onto the anode surface by screen printing, followed by sintering at 300 °C for 2 h to obtain the catalyst wrapped anode supporting cells with a structure of Ru/CZ4–Ni | Ni–BCZYYb | BCZYYb | LSCF–BCZYYb (Ru/CZ4 cell). The main steps of the above procedure are shown in follow scheme.

**
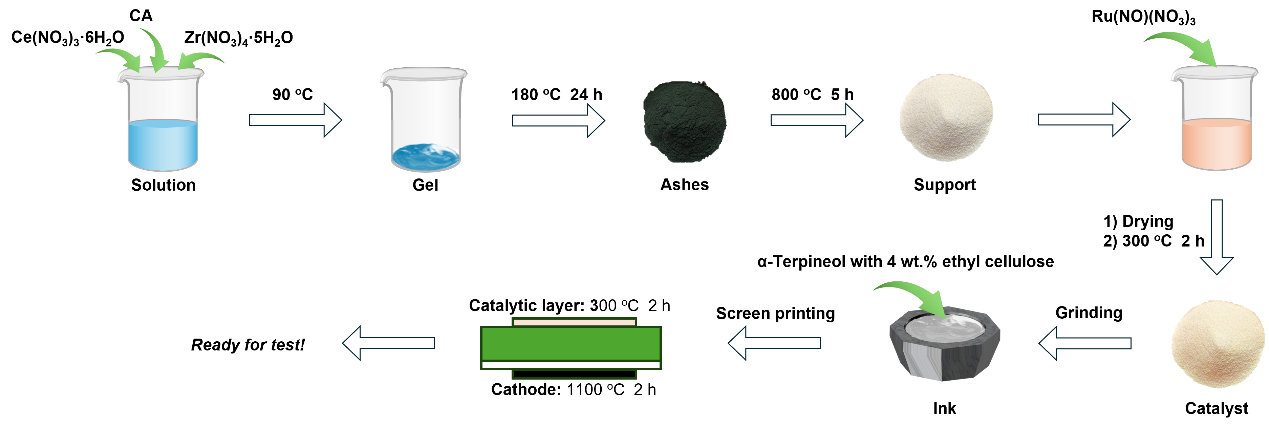
**

**Scheme 1.** The preparation of Ru/CZ4 and of the Ru/CZ4 cell.

**1.3 Measurements and characterization**

**1.3.1 Electrochemical Measurements**

Impedance spectroscopy and electrochemical performance tests were conducted using a Zahner IM–6 electrochemical workstation (Germany). The assembly process of the flat–plate battery reaction tube is as follows: (1) Silver paste used for the current collection was coated on the cathode surface of the button cell by screen printing. (2) A 50 mm silver wire, a 50 mm platinum wire, a 2×2 mm² silver mesh, and a 300–mesh nickel foam with a diameter of 15 mm were prepared and assembled on the alumina tube with the order of silver wire–nickel foam–button cell (anode facing down)–silver mesh–platinum wire. The silver wire was coated with platinum paste to enhance conductivity. (3) Finally, the alumina tube mounted button cell was sealed with ceramic adhesive and aged at room temperature for 12 h until the adhesive solidified. Before the tests, the reaction tube was placed in a tube furnace and heated to 650 °C with a rate of 3 °C/min, and pretreated high–purity H_2_ was introduced into the tube for 30–60 minutes. The tests could begin until the OCV of cell stabilized. The Mott–Schottky tests for the anode catalysts were carried out at the Zahner IM–6 electrochemical workstation, with a voltage range of –1 to 1 V and frequencies of 1500 Hz, 1000 Hz, and 500 Hz. Ag/AgCl electrode was used as the reference electrode. The conductivities of the anode catalysts were obtained through a four–probe method, in which the samples were connected to the Keithley 2400 digital source meter via Pt wire with the four–electrode resistance testing mode. H_2_ was introduced at a flow rate of 50 mL min^–1^ during the tests, and the resistance values were measured and recorded in the temperature range of 500–700 °C (with an interval of 50 °C).

**1.3.2 Material Characterization**

X–ray diffraction (XRD) was conducted on a Panalytical X’Pert Pro diffractometer equipped with Cu Kα radiation (λ = 1.5418 Å, 45 kV, and 40 mA). The scanning rate was 5°/min, with a continuous scan range of 10°–90° (2θ). The unit cell parameters were calculated using Unit Cell software. Raman spectroscopies were performed using an OPTIMA 8000 micro confocal Raman spectrometer (Renishaw, UK) with a 532 nm Ar ion laser. The test center was 1000 cm^–1^ with an exposure time of 3 seconds for each scan (3 replicates), and the laser intensity was controlled at 10%. The scanning electron microscope (SEM) images for the cross–section of cells was obtained by a S–4800 high–resolution scanning electron microscope (Hitachi, Japan). The transmission electron microscope (TEM) images of different samples were obtained through JEM–F200 high–resolution transmission electron microscopy (HR–TEM), and the corresponding crystal planes were determined using Digital Micrograph software. Additionally, energy–dispersive X–ray spectroscopy (EDX) was employed to obtain information of the elemental distribution of the anode surface. H_2_ temperature–programmed reduction (TPR) and O₂ pulse chemisorption were conducted using an Auto Chem II 2920 chemisorption analyzer (Micromeritics, USA). Prior to oxygen adsorption measurements, the catalyst sample pretreatment consisted of purging the sample with an inert gas stream (He/Ar, 30 mL/min) while heating the sample to 400 °C at a controlled ramp rate (5 °C/min) and maintaining it at this temperature for 1 hour to ensure complete removal of physically adsorbed water and surface contaminants. Pulses of pure O₂ (30 mL at a time) were injected through a six-way valve until the TCD detection signal was saturated. The adsorption volume is calculated as follows:

$$\text{N}\text{(}\text{O}_{\text{2}}\text{)}\text{=}\sum{\text{(}\text{A}}_{\text{injected}}\text{−}\text{A}_{\text{eluted}}\text{)}\text{×}\frac{\text{C}_{\text{O}_{\text{2}}}}{\text{A}_{\text{calibration}}}$$

The UV–Vis diffuse reflectance spectroscopy were conducted using a Lambda 950 UV–Vis diffuse reflectance spectrometer (Perkin Elmer, USA). The ammonia decomposition performance tests were carried out using a fixed–bed reactor at atmospheric pressure and analyzed with a Fuli GC9790 Plus gas chromatograph.

1.3.3 Ammonia decomposition activity test

Ammonia decomposition activity was evaluated using a fixed bed catalyst evaluation device. Prior to the test, 0.1 g of catalyst (40~60 mesh) was placed in a reaction tube, and the catalyst was reduced at 500 °C for 3 h in a 50% H_2_/Ar atmosphere (50 mL min^-1^), followed by purging for 1 h in an Ar atmosphere (50 mL min^-1^), and finally the activity was evaluated in an NH_3_ atmosphere (25 mL min^-1^ or 50 mL min^-1^). All activity tests of the catalysts were carried out at atmospheric pressure in the temperature range of 450-750 °C with a reaction headspace velocity (GHSV) of 30,000 mL g_cat_^-1^ h^-1^. The concentration of NH_3_ and N_2_ in the outlet gas were analyzed online using a GC 9790 Plus gas chromatograph equipped with a thermal conductivity detector, with hydrogen as the carrier gas. The ammonia conversion(*X*_NH3_) was calculated as follow：

$$X_{NH3}\text{=}\frac{V_{N2}/V_{NH3}}{{(V}_{N2}/V_{NH3}\text{)+0.5}}\text{ }$$

Here, *V*_N2_ and *V*_NH3_ represent the volume fractions of N_2_ and NH_3_ in the outlet gas, respectively.

The H_2_ formation rate is then obtained by the following equation:

$$\text{H}\text{2}\text{ formation rate(mmol·}{\text{g}_{\text{cat}}}^{\text{-1}}\text{·mi}\text{n}^{\text{-1}}\text{) =}\frac{\frac{\text{V}\text{NH3}}{\text{22.4}}\text{×}X_{NH3}\text{×1.5}}{\text{m}\text{cat}}$$

**1.4** **Theoretical power efficiency calculation**

The theoretical power efficiency (η_th_) can be calculated from the following expression:

$$\text{η}_{\text{th}}\text{=ΔG/ΔH}$$

Where ΔG and ΔH is the change of Gibbs free energy and the change of enthalpy, respectively. The thermodynamic data ΔG and ΔH at different temperatures are obtained from previous report [1]. The thermal values and the η_th_ of for H_2_ fuel cell and NH_3_ fuel cell are in reported Table S1.

**Supplementary figures**


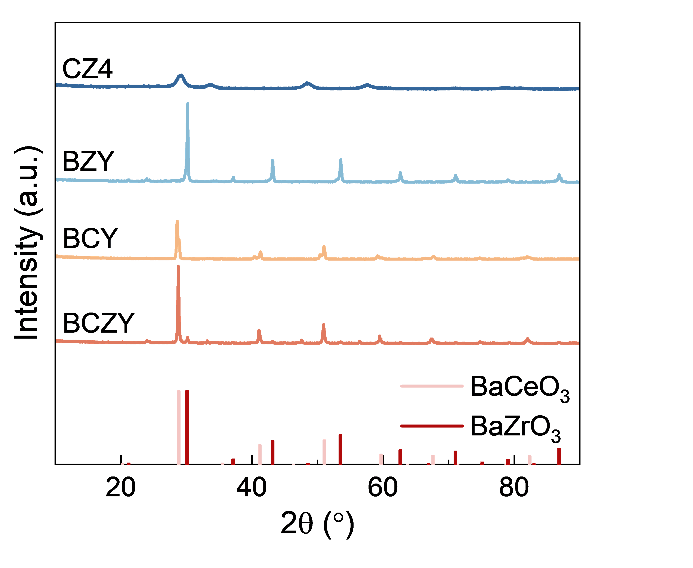


**Fig. S1.** XRD spectra of various catalyst support.


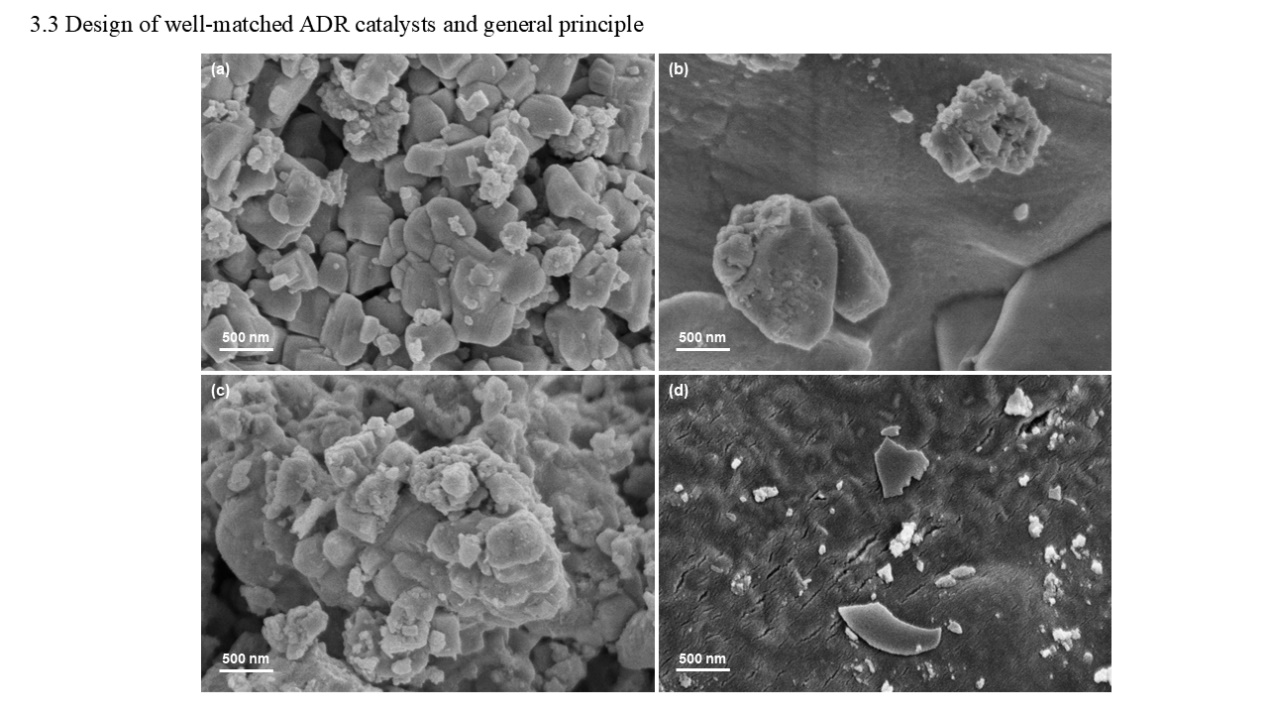


**Fig. S2.** SEM images of various catalysts: **a.** Ru/BZY, **b.** Ru/CZY, **c.** Ru/BCZY and **d.** Ru/CZ4.


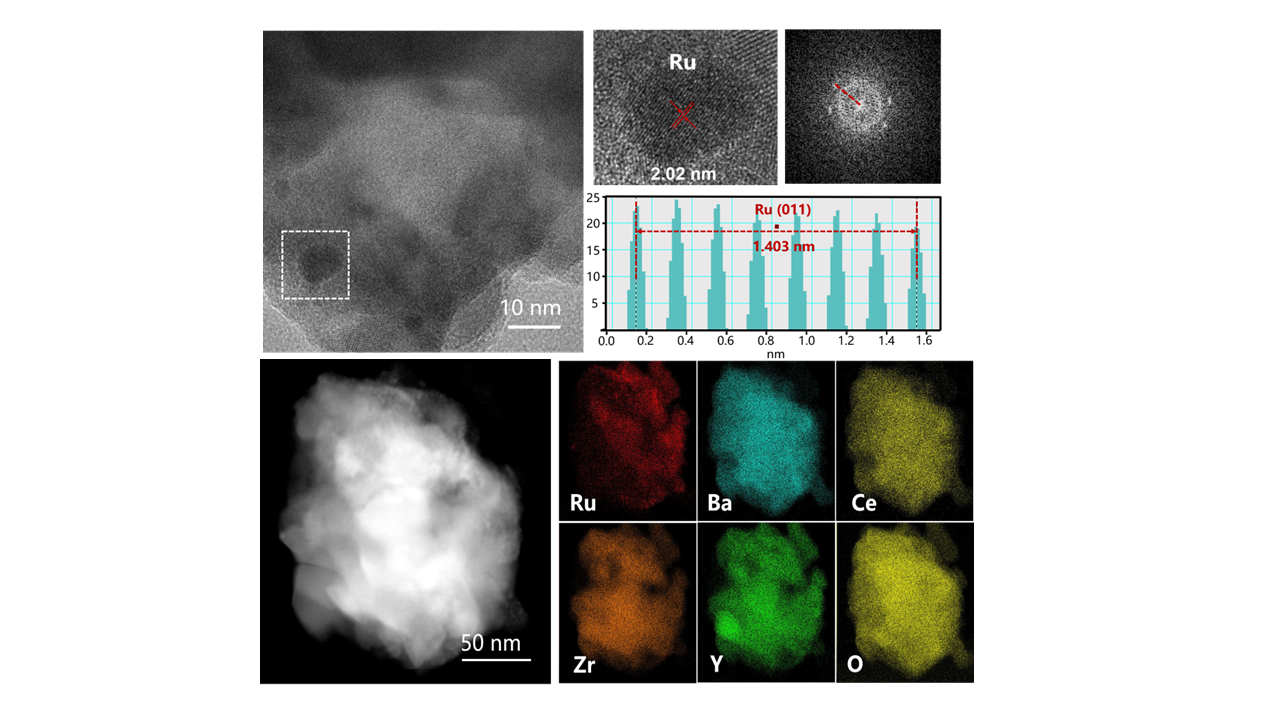


**Fig. S3.** HR–TEM images and a measurement of ruthenium particle size, STEM image and EDX elemental mapping of the Ru/BCZY catalyst.


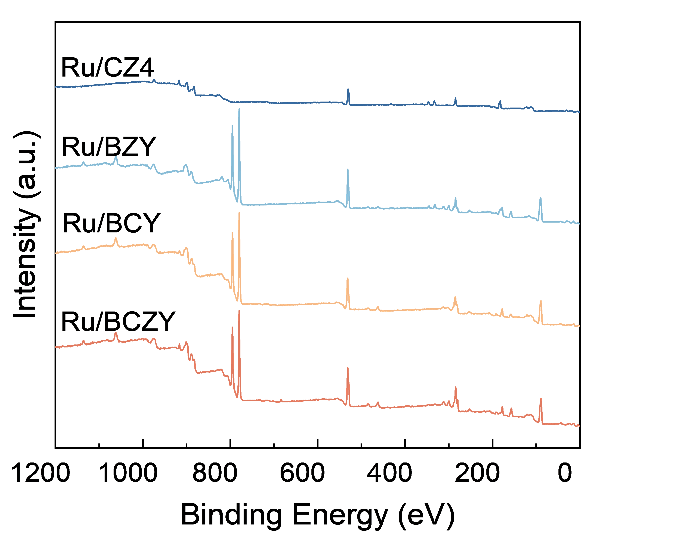


**Fig. S4.** XPS survey of Ru–based catalysts


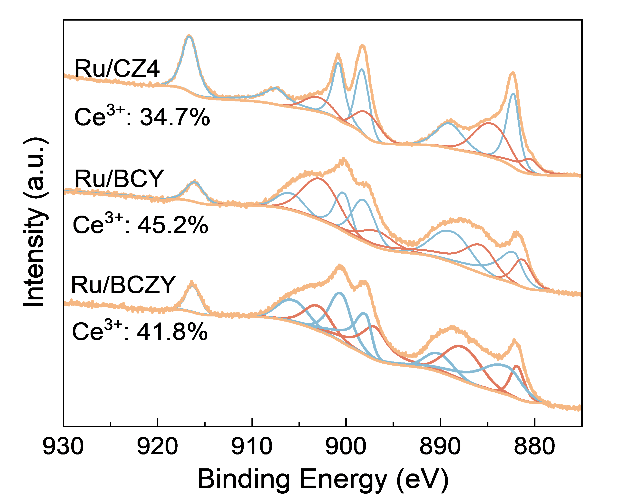


**Fig. S5.** Ce 3d XPS spectra of Ru–based catalysts.


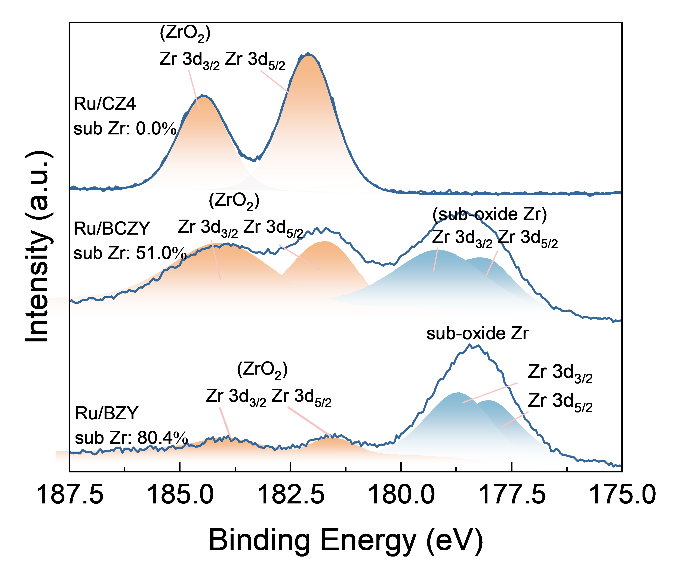


**Fig. S6.** Zr 3d XPS spectra of Ru–based catalysts.


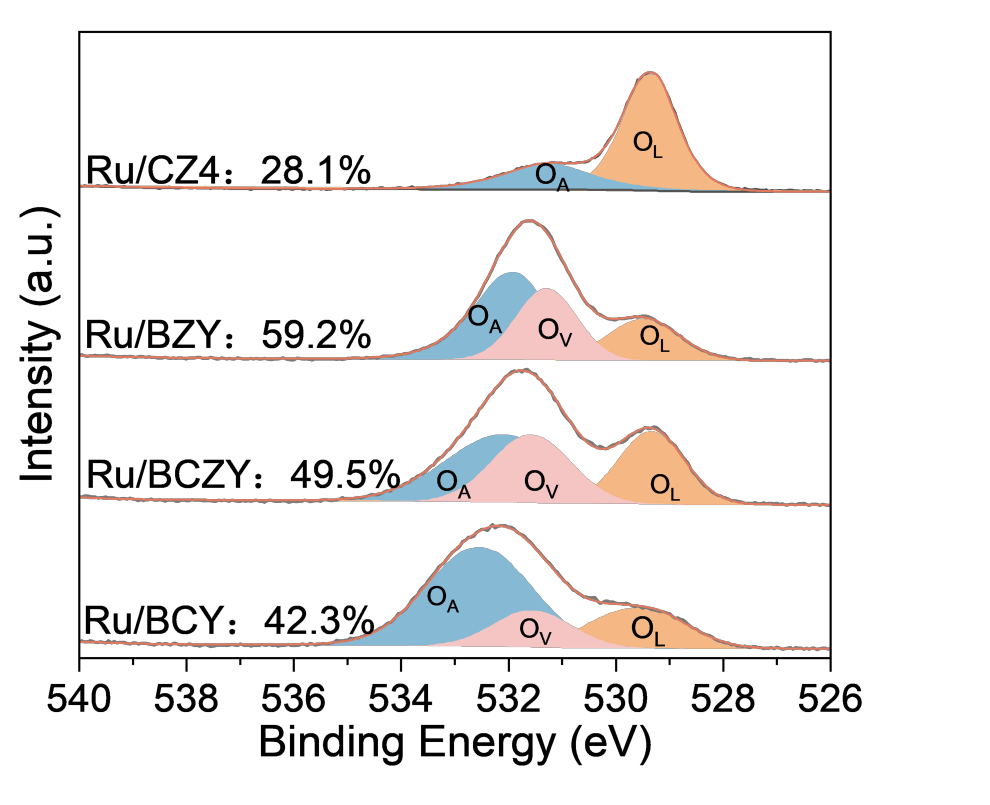


**Fig. S7.** O 1s XPS profiles of the Ru/BZY, Ru/BCZY, Ru/BZY and Ru/CZ4 catalysts.


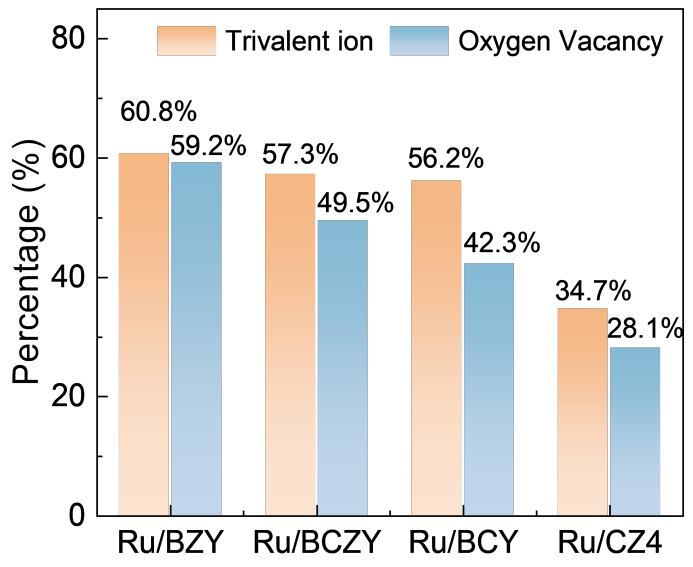


**Fig. S8.** Trivalent ion and oxygen vacancy content of the Ru/BZY, Ru/BCZY, Ru/BZY and Ru/CZ4 catalysts.


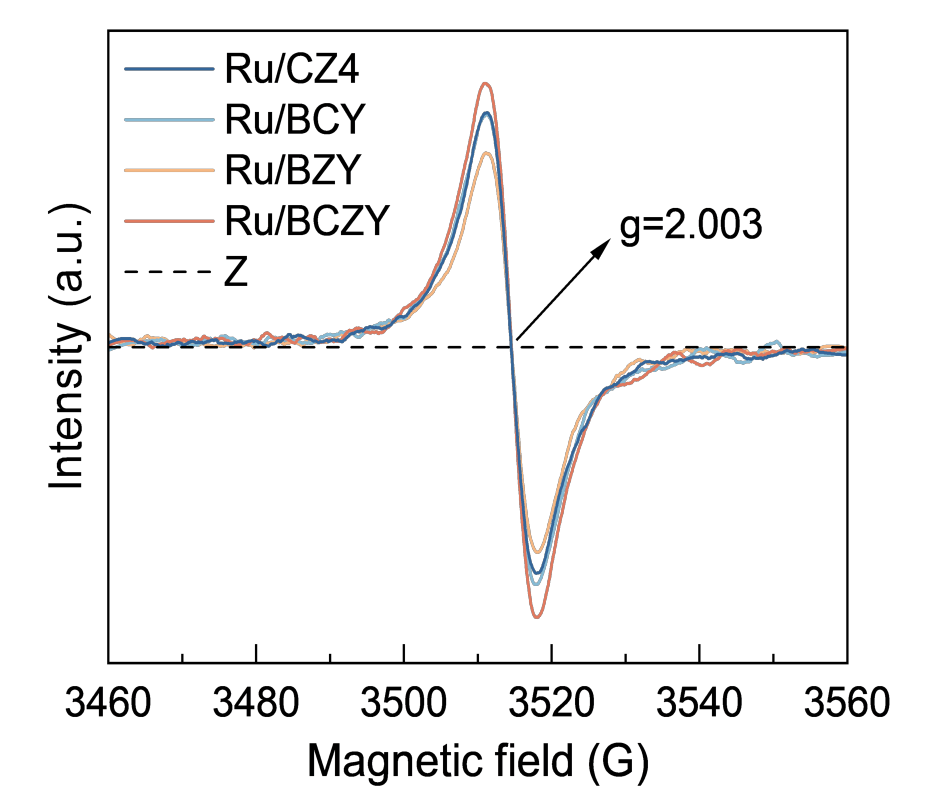


**Fig. S9.** EPR spectra for Ru/BZY, Ru/BCY, Ru/BCZY and Ru/CZ4.


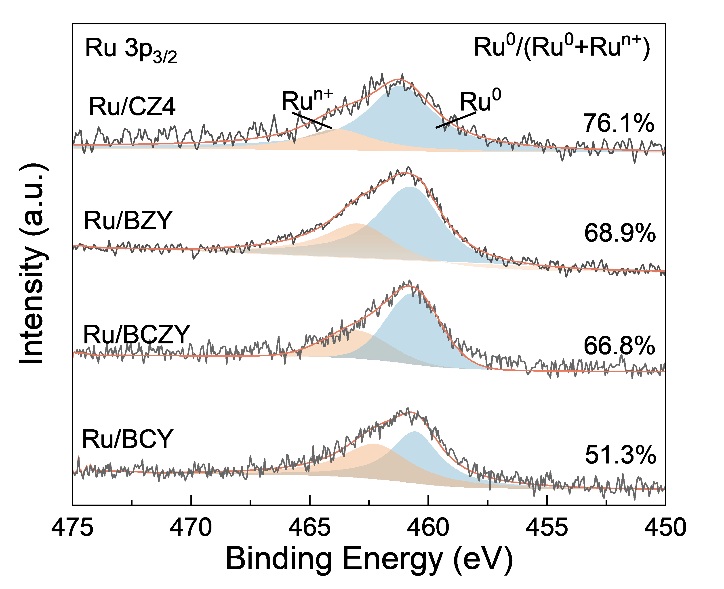


**Fig. S10.** Ru 3p XPS spectra of Ru–based catalysts


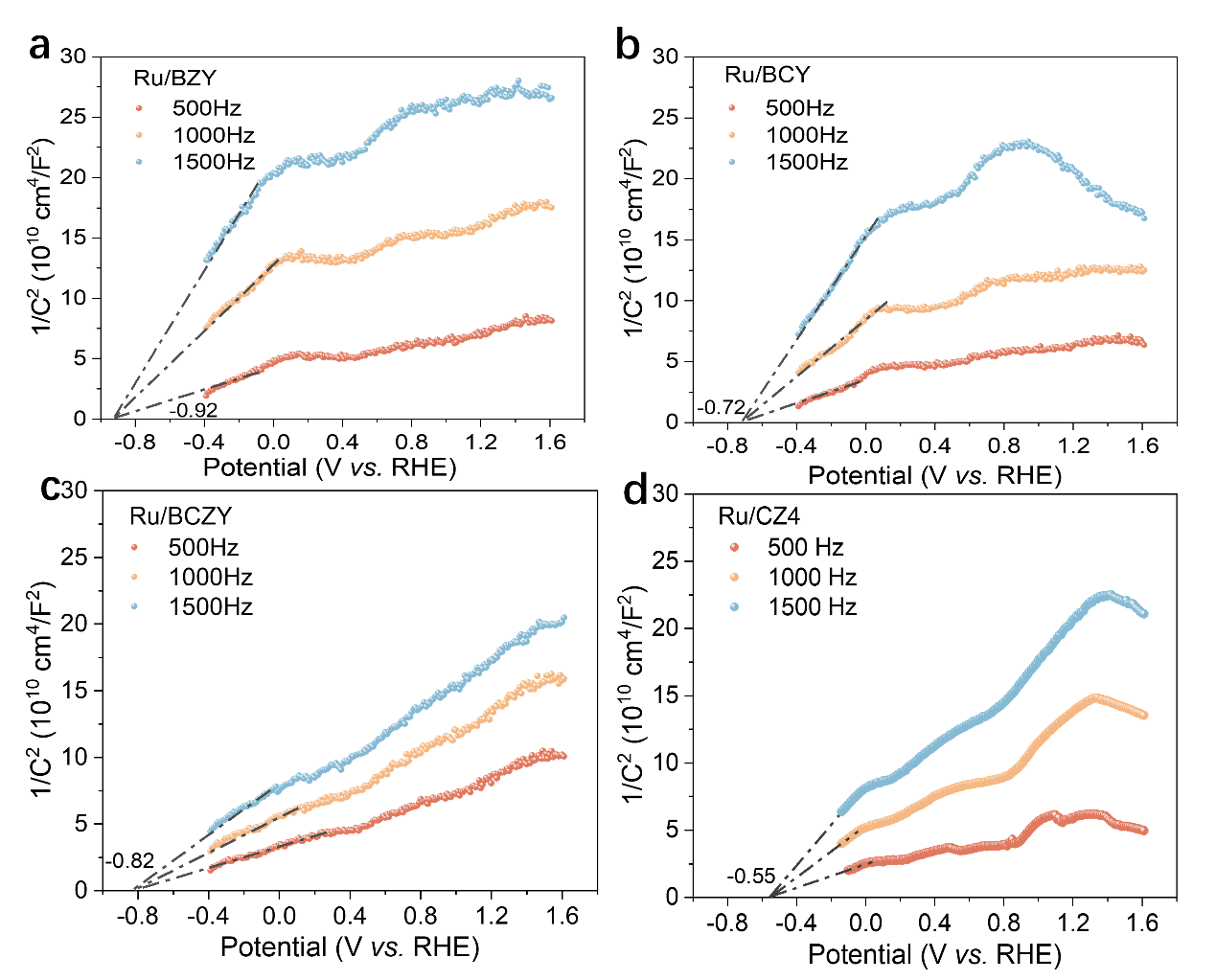


**Fig. S11.** Mott–Schottky curves of the **a.** Ru/BZY, **b.** Ru/BCY, **c.** Ru/BCZY and **d.** Ru/CZ4.


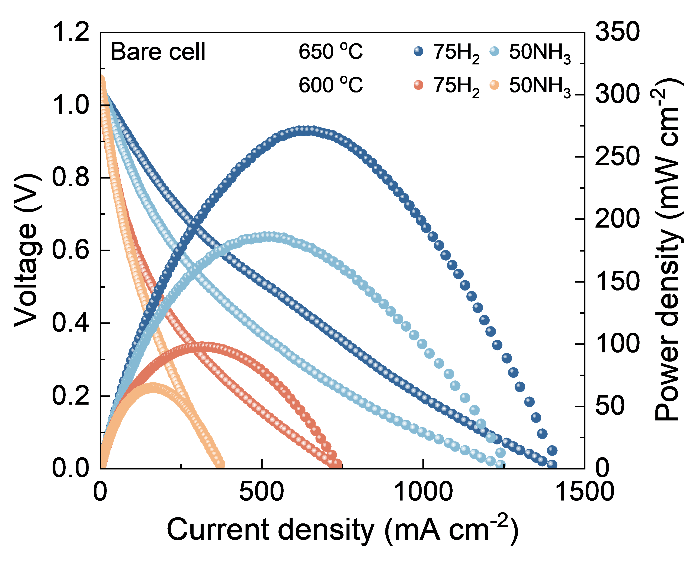


**Fig. S12.** I–V–P curves of bare cell under different temperatures.


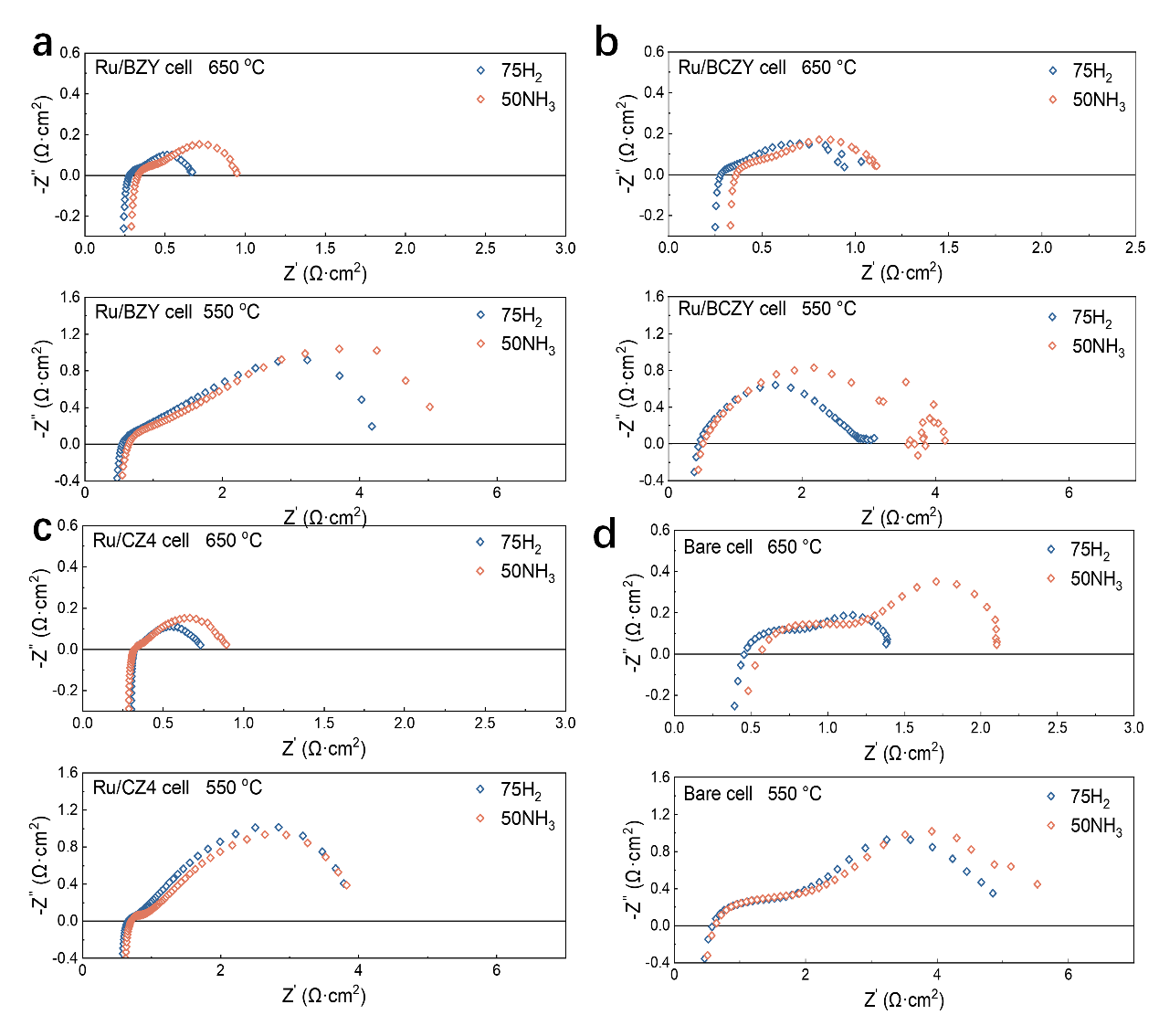


**Fig. S13.** EIS spectrum of **a.** Ru/BZY, **b.** Ru/BCZY, **c.** Ru/CZ4, **d.** bare cell


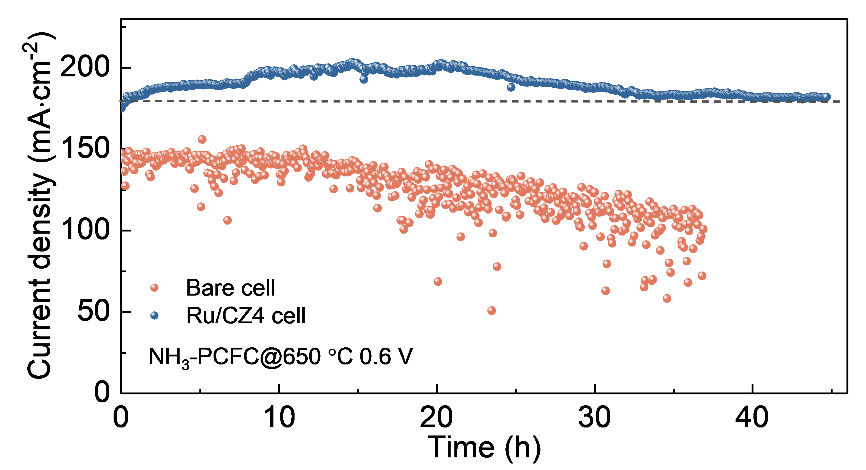


**Fig. S14.** Stability test of Ru/CZ4 cell and bare cell at 0.6 V and 650 ℃.


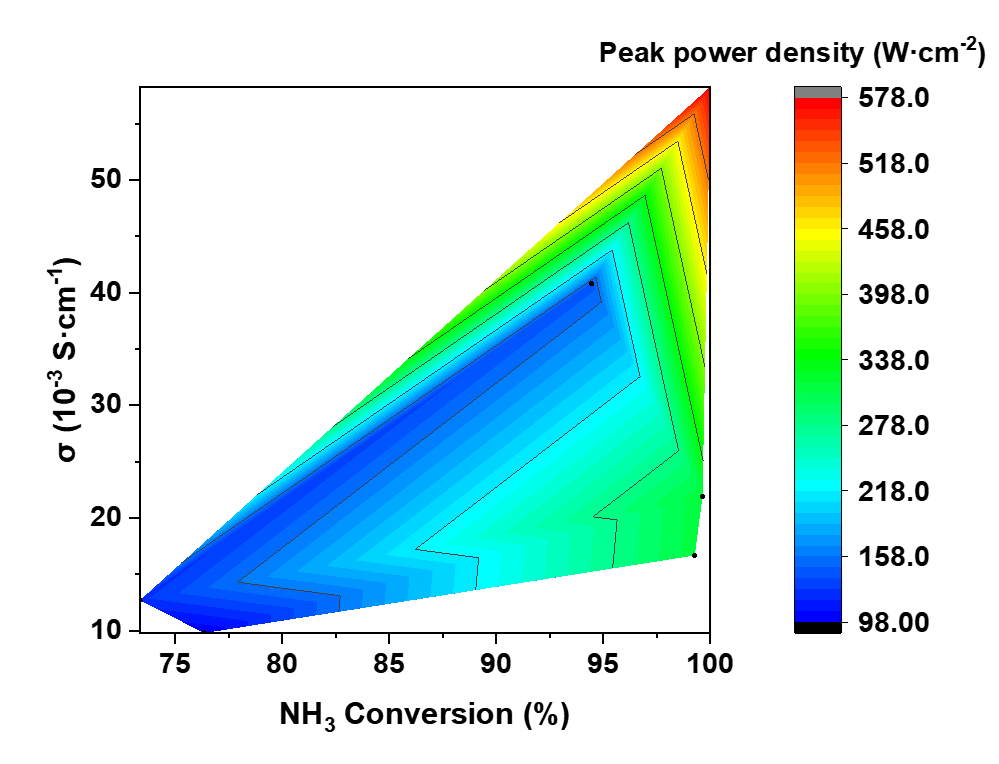


**Fig. S15.** Comparison of PPDs of NH_3_-PCFCs with catalyst layers affected by electrical conductivities and ammonia decomposition activity.

**Supplementary tables**

Table S1. Thermal values and FC efficiency at different temperature.

|  |  |  | H_2_ fuel cell | | | NH_3_ fuel cell | | |
| --- | --- | --- | --- | --- | --- | --- | --- | --- |
|  | T  (K) | T  (℃) | ΔG  (kJ/mol) | ΔH  (kJ/mol) | FC eff.  (%). | ΔG  (kJ/mol) | ΔH  (kJ/mol) | FC eff.  (%) |
| Liquid H_2_O | 298.15 | 25.00 | -237.14 | -285.83 | 82.97 | -355.71 | -382.81 | 92.92 |
|  | 300.00 | 26.85 | -236.84 | -285.77 | 82.88 | -355.26 | -382.68 | 92.84 |
|  | 373.15 | 100.00 | -225.05 | -283.46 | 79.39 | -337.57 | -377.68 | 89.38 |
| Gaseous H_2_O | 373.15 | 100.00 | -225.18 | -242.58 | 92.83 | -337.77 | -377.68 | 89.43 |
|  | 400.00 | 126.85 | -223.95 | -242.85 | 92.22 | -335.92 | -316.19 | 106.24 |
|  | 500.00 | 226.85 | -219.11 | -243.83 | 89.86 | -328.67 | -315.84 | 104.06 |
|  | 600.00 | 326.85 | -214.08 | -244.76 | 87.47 | -321.12 | -315.72 | 101.71 |
|  | 700.00 | 426.85 | -208.90 | -245.63 | 85.04 | -313.35 | -315.79 | 99.23 |
|  | 800.00 | 526.85 | -203.60 | -246.45 | 82.61 | -305.39 | -316.00 | 96.64 |
|  | 900.00 | 626.85 | -198.19 | -247.19 | 80.18 | -297.29 | -316.33 | 93.98 |
|  | 1000.00 | 726.85 | -192.71 | -247.86 | 77.75 | -289.07 | -316.74 | 91.26 |
|  | 1100.00 | 826.85 | -187.17 | -248.46 | 75.33 | -280.75 | -317.21 | 88.51 |
|  | 1200.00 | 926.85 | -181.57 | -249.00 | 72.92 | -272.36 | -317.72 | 85.72 |
|  | 1300.00 | 1026.85 | -175.93 | -249.48 | 70.52 | -263.90 | -318.26 | 82.92 |
|  | 1400.00 | 1126.85 | -170.26 | -249.89 | 68.13 | -255.39 | -318.82 | 80.11 |

**References**

1. Lide DR. CRC handbook of chemistry and physics. 84th Edition CRC Press, 2003.
